# Clinical and biological heterogeneities in triple-negative breast cancer reveals a non-negligible role of HER2-low

**DOI:** 10.1186/s13058-023-01639-y

**Published:** 2023-03-30

**Authors:** Xi′e Hu, Ping Yang, Songhao Chen, Gang Wei, Lijuan Yuan, Zhenyu Yang, Li Gong, Li He, Lin Yang, Shujia Peng, Yanming Dong, Xianli He, Guoqiang Bao

**Affiliations:** 1 Department of General Surgery, The Second Affiliated Hospital of Air Force Medical University, No. 569, Xinsi Road, Baqiao District, Xi’an, Shannxi China; 2 Department of Pathology, The Second Affiliated Hospital of Air Force Medical University, No. 569, Xinsi Road, Baqiao District, Xi’an, Shannxi China; 3 Department of Pathology, The University of Oklahoma Health Sciences Center, Oklahoma City, USA

**Keywords:** Triple-negative breast cancer, HER2-low, Single-cell RNA sequencing, Prognosis, Heterogeneity, Tumor microenvironment

## Abstract

**Background:**

HER2-low could be found in some patients with triple-negative breast cancer (TNBC). However, its potential impacts on clinical features and tumor biological characteristics in TNBC remain unclear.

**Methods:**

We enrolled 251 consecutive TNBC patients retrospectively, including 157 HER2-low (HER2_low_) and 94 HER2-negtive (HER2_neg_) patients to investigate the clinical and prognostic features. Then, we performed single-cell RNA sequencing (scRNA-seq) with another seven TNBC samples (HER2_neg_ *vs.* HER2_low_, 4 *vs.* 3) prospectively to further explore the differences of tumor biological properties between the two TNBC phenotypes. The underlying molecular distinctions were also explored and then verified in the additional TNBC samples.

**Results:**

Compared with HER2_neg_ TNBC, HER2_low_ TNBC patients exhibited malignant clinical features with larger tumor size (*P* = 0.04), more lymph nodes involvement (*P* = 0.02), higher histological grade of lesions (*P* < 0.001), higher Ki67 status (*P* < 0.01), and a worse prognosis (*P* < 0.001; HR [CI 95%] = 3.44 [2.10–5.62]). Cox proportional hazards analysis showed that neoadjuvant systemic therapy, lymph nodes involvement and Ki67 levels were prognostic factors in HER2_low_ TNBC but not in HER2_neg_ TNBC patients. ScRNA-seq revealed that HER2_low_ TNBC which showed more metabolically active and aggressive hallmarks, while HER2_neg_ TNBC exhibited signatures more involved in immune activities with higher expressions of immunoglobulin-related genes (*IGHG1*, *IGHG4*, *IGKC*, *IGLC2*); this was further confirmed by immunofluorescence in clinical TNBC samples. Furthermore, HER2_low_ and HER2_neg_ TNBC exhibited distinct tumor evolutionary characteristics. Moreover, HER2_neg_ TNBC revealed a potentially more active immune microenvironment than HER2_low_ TNBC, as evidenced by positively active regulation of macrophage polarization, abundant CD8^+^ effector T cells, enriched diversity of T-cell receptors and higher levels of immunotherapy-targeted markers, which contributed to achieve immunotherapeutic response.

**Conclusions:**

This study suggests that HER2_low_ TNBC patients harbor more malignant clinical behavior and aggressive tumor biological properties than the HER2_neg_ phenotype. The heterogeneity of HER2 may be a non-negligible factor in the clinical management of TNBC patients. Our data provide new insights into the development of a more refined classification and tailored therapeutic strategies for TNBC patients.

**Supplementary Information:**

The online version contains supplementary material available at 10.1186/s13058-023-01639-y.

## Introduction

Breast cancer (BC) is the most frequently diagnosed tumor in women worldwide, and it remains as the second leading cause of cancer-related death [1]. Triple-negative BC (TNBC) accounts for 15–20% of all BC cases which is usually defined as the absence of estrogen receptor (ER), progesterone receptor (PR) and human epidermal growth factor receptor 2 (HER2) [2]. TNBC is an intertwined disease characterized by its early onset, increased metastatic risk and poor prognosis [3–5]. It exhibits highly clinical and molecular inherent heterogeneity consisting of different intron subtypes with distinguishing biological characteristics, treatment sensitivities and clinical outcomes [6, 7]. Therefore, the lack of therapeutic targets and its malignant biological features render it a challenging issue in the clinical management of BC.

The difference in HER2 expression levels is one of the apparent heterogeneous properties of TNBC which can be assessed in clinical practice [8]. According to pathological assessments of HER2 expression levels by immunohistochemistry (IHC) and fluorescence in situ hybridization (FISH), TNBC can be divided into two different categories: HER2-negative/HER2-zero TNBC (HER2_neg_ TNBC; IHC 0) and HER2-low TNBC (HER2_low_ TNBC; IHC1 + , or IHC2 + and FISH-negative). Traditionally, this differentiation seems to be less pivotal for patients’ clinical treatment options, but recent studies have suggested a potential efficacy of novel HER2-targeted antibody drug conjugates (ADCs) in the treatment of HER2_low_ BC [9, 10], which opens up an emerging era for evaluating the implicit role of HER2-low in the clinical setting of TNBC.

To date, several studies have investigated the clinical features and biological hallmarks of HER2_low_ BC [8, 11–17], which highlight the potential effect of HER2-low on the treatment response and clinical outcomes of TNBC. However, current studies on the significance of HER2-low are still inconclusive [8, 13, 15–17], and the underlying pathogenic role of HER2-low in TNBC is less explored. Furthermore, the potential biological differences between HER2_low_ and HER2_neg_ TNBC categories also remain poorly understood. Therefore, a more comprehensive analysis of the biological influence of HER2-low on TNBC is urgently needed, which may lay a foundation for future TNBC target-based therapy, especially in the new era of precision therapy.

Single-cell RNA sequencing (scRNA-seq) is a promising technique for defining tumor subpopulations and identifying potential treatment targets [7] and has a potential to detect subtle discrepancies between different tumor subtypes for TNBC. Hence, we employed this technique to better understand the underlying features of HER2_low_ TNBC and HER2_neg_ TNBC.

In this study, we investigated the clinical characteristics between 157 HER2_low_ TNBC and 94 HER2_neg_ TNBC patients; then, scRNA-seq was prospectively performed on 36,168 cells from three HER2_low_ and four HER2_neg_ TNBC patients for the further in-deep exploration. The aims of this study were as following: a) to investigate the clinical significance of HER2-low in TNBC patients; b) to delineate the transcriptome patterns of HER2_low_ and HER2_neg_ TNBC; and c) to explore the potential impacts of HER2 status on tumor behaviors and microenvironment properties in TNBC. This study contributes to a better understanding of the potential clinical and biological heterogeneities of HER2_low_ and HER2_neg_ TNBC and may provide novel clues for the development of more refined classification and tailored therapeutic strategies for TNBC.

## Methods

### Clinical patients and sample collection

**Fig. 1:**
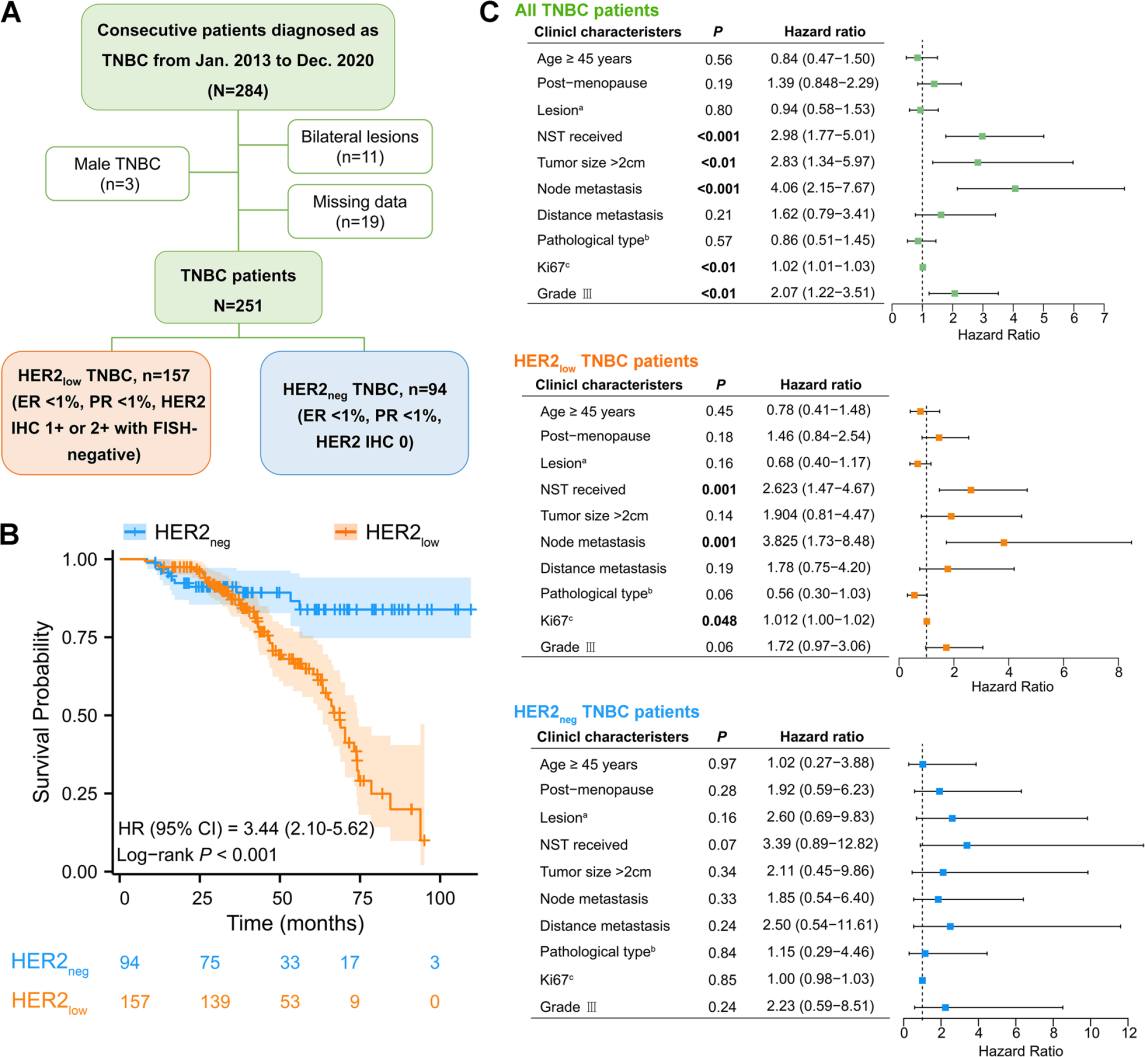
Investigations of clinical features in TNBC patients with different HER2 status. (**A**) Flow chart of study population enrollment. (**B**) Overall survival curves of HER2_low_ and HER2_neg_ TNBC patients diagnosed at our institution in the past seven years, using logrank test. *P* < 0.05 indicates statistical significance. The detailed survival data are attached in Additional file 8: Table S1. (**C**) Forest plots showing the prognostic effects of clinical features in all TNBC, HER2_low_, and HER2_neg_ TNBC patients, respectively

To investigate the clinical characteristics of TNBC with different HER2 status, a total of 251 female patients consecutively confirmed as TNBC pathologically in our institution from Jan. 2013 to Dec. 2020 were incorporated in this study, including 157 HER2_low_ and 94 HER2_neg_ TNBC patients. The clinicopathological data were retrospectively collected from medical records of the institutional database. In this study, all the determinations of ER, PR, and HER2 status were performed according to the ASCO/CAP guidelines [18, 19]. Briefly, the TNBC lesion was defined as ER < 1%, PR < 1%, and HER2 IHC 0 or IHC 1 + /2 + with a negative result of HER2 amplification by FISH. HER2 IHC 1 + and 2 + (FISH-negative) were considered as HER2-low. The overall survival was defined as the time from TNBC diagnosis to the time of the last follow-up time (Jan 1^st^, 2021), the time of death or the time lost to follow-up. The follow-up data of the patients were collected based on medical records or telephone interviews. Males, patients who refused follow-up, or patients with missing clinicopathological data were excluded from this study. The detailed inclusion and exclusion process of patients is shown in Fig. 1A, and the detailed data of the patients are shown in Additional file 8: Table S1.

The patient recruitment time for scRNA-seq was from Jan. 2021 to Jan. 2022, and all patients included provided with written informed consent. Seven enrolled patients with pathologically confirmed TNBC were all female, range 38 to 49 years old and not pregnant. All enrolled patients were treatment-naïve with a unilateral lesion and consented to receive ultrasound-guided pathological puncture. Notably, P3, P4 and P5 were HER2_low_ TNBC, and P1, P2, P6 and P7 were HER2_neg_ TNBC (Fig. 1A). P1, P2 and P7 received anti-PD-1 immunotherapy (Tislelizumab Injection from BeiGene, Ltd) combined with chemotherapy (nab-paclitaxel) as neoadjuvant systematic therapy (NST) every three weeks (Q3W) for four cycles. Finally, 7 treatment-naïve puncture samples and 3 postoperative specimens of P1, P2 and P7 (after NST) were obtained. The basic demographic characteristics, clinical profiles and sampling information of the patients are presented in Additional file 9: Table S2.

This prospective study was conducted under a protocol approved by the Institutional Ethics Committee of The Second Affiliated Hospital of Air Force Medical University (No. K202010-04) and in accordance with the Declaration of Helsinki.

### Tissue dissociation and preparation

Fresh breast tissues were stored in Tissue Preservation Solution (Singleron Biotechnologies, Nanjing, China) and placed on ice after the biopsy within 30 min. The specimens were washed three times and then cut into slices of 1 to 2 mm. Subsequently, the tissue pieces were digested in a 15 ml centrifuge tube at 37 °C with continuous agitation for 15 min. Then, they were centrifuged at 500 × g for 5 min and suspended gently with PBS (HyClone, USA). Finally, the samples were stained with trypan blue (Sigma, USA), and cell viability was evaluated under a phase-contrast microscope (Nikon, Japan).

### Library preparation

Single-cell suspensions (1 × 10^5^ cells/ml) with PBS (HyClone, USA) were loaded into microfluidic devices using the Singleron Matrix® Single Cell Processing System (Singleron). Subsequently, the scRNA-seq library was established according to the protocol of the GEXSCOPE® Single Cell RNA Library Kit (Singleron) [20]. Libraries for individuals were diluted to 4 nM and pooled for sequencing. Finally, the pools were sequenced with 150 bp paired-end reads on an Illumina HiSeq X instrument.

### Quality control and pre-processing

Cells were filtered by gene counts between 200 and 5,000 and unique molecular identifier (UMI) counts below 30,000. Consistent with previous studies [21–24], we removed the cells with over 50% mitochondrial content in order to optimize keeping cells and removing dead and dying cells. After filtering, we used functions from Seurat v3.1.2 for dimension reduction and clustering. The raw reads were processed to generate gene expression profiles. Briefly, the cell barcode and UMI were extracted after filtering read one without poly-T tails. We trimmed (fastp V1) the adapters and poly-A tails before aligning read two to GRCh38 with Ensemble v92 gene annotation (fastp 2.5.3a and featureCounts 1.6.2) [25]. Reads of the same cell barcode, UMIs and genes were combined to count the number of UMIs in each cell. UMI count tables in each cell barcode were applied for further analysis.

### Dimensionality reduction

The Read10 × function was applied to process the Seurat object with individual gene expression data. For each sample, gene expression was expressed as a fraction of the genes, which were then multiplied by 10,000. These genes were converted into natural logarithms after the addition of 1 and normalized to avoid obtaining logarithms of 0. Before we performed principal component analysis (PCA) based on these standardized expression matrices, we identified the top 3000 highly variable genes (HVGS) from the standardized expression matrix and concentrated and scaled them. The batch effects were removed by the Harmony package (version 1.0) of R/Rstudio software (version 3.6.1) based on the identity of the top 50 PCA components [26].

### scRNA-seq-based copy number variation (CNV) detection

The InferCNV package [27] was used to detect CNV in malignant breast cells. Non-malignant breast cells were identified as the baseline to evaluate the CNVs of malignant cells. Genes expressed in more than 20 cells were sorted according to their loci on each chromosome. The relative expression value was centered at 1, and a total of 1.5 standard deviations from the residual standardized expression value were considered the ceiling. The sliding window size of 101 genes was used to smooth the relative expression of each chromosome to eliminate the influence of gene-specific expression.

### Intratumoral heterogeneity (ITH) score calculation

The ITH score was calculated by the algorithm described in BC. The ITH score was defined as the average Euclidean distance between the individual cells and all other cells, according to the first 20 principal components derived from the normalized expression levels of highly variable genes. The highly variable gene was identified by the Seurat package with the default parameters.

### Analysis of differential expression genes (DEGs) and cell type annotations

Genes expressed in more than 10% of the cells in a cluster and with an average log (fold change) of greater than 0.25 were selected as DEGs by Seurat v3.1.2 based on the Wilcox likelihood-ratio test with default parameters.

Cell type identification and clustering analysis were performed by the Seurat program [28, 29]. Furthermore, the Seurat program (http://satijalab.org/seurat/) was applied for the analysis of RNA-seq data. UMI count tables were loaded into R by “read.table” function. Afterward, the parameter resolution to 2.0 was set for the “Find Clusters” function for clustering analyses. For subclustering of various cell types, we set the resolution at 1.2. The UMAP algorithm was used to visualize cells in a two-dimensional space. The cell type of each cluster was identified according to the expression of typical markers in DEGs using the SynEcoSys database. Doublet cells were mainly judged based on marker gene expression, which would commonly express marker genes of two or more cell types that already exist on the clustering map and have no differentiation relationship. The doublet gene expression profile may affect the results of cell subtype clustering, cell differentiation status analysis, as well as cell subtype functional enrichment analysis, resulting in biased understanding of the biological significance [30]. Therefore, annotated doublet is generally removed in this study to reduce the possibility of errors in subsequent analysis. Detailed information on the cell markers is shown in Additional file 10: Table S3.

### Pathway enrichment analysis

To investigate the potential functions of DEGs, Gene Ontology (GO) and Kyoto Encyclopedia of Genes and Genomes (KEGG) analyses were applied with the “ClusterProfiler” R package 3.6.1. In this study, the gene sets in the “biological process (BP)” of GO pathway were mainly considered. GO and KEGG functional enrichment analyses were conducted to explore biological functions or pathways significantly associated with the specifically expressed genes [31]. Gene set enrichment analysis (GSEA) was performed on genes expressed in tumor clusters. For GSVA pathway enrichment analysis, the average gene expression of each tumor cell in every TNBC group was used as input data using the GSVA package.

### Trajectory analysis

To map the differentiation of tumor cells in the two TNBC groups, pseudotime trajectory analysis was performed by Monocle v2 [32]. To construct the trajectory, the highly variable genes were selected from tumor clusters 1 to 15 via the Seurat v3.1.2 Find Variable Features function. The dimension reduction was performed by DDRTree. The trajectory was subsequently visualized and the dynamic changes in gene expression over pseudotime were displayed. The differentiation status of each tumor cell subcluster was detected by CytoTRACE [33].

### Single-cell T-cell receptor-sequencing (scTCR-seq)

scTCR-seq libraries were constructed according to the protocol of the GEXSCOPE Single Cell Immuno-TCR Kit (Singleron Biotechnologies). Briefly, the magnetic beads with molecular labels captured the poly-A tail of mRNAs and TCR region of immune cells after the cells were lysed. Subsequently, the magnetic beads in the chip were collected, and then mRNAs captured by the magnetic beads were reverse transcribed into complementary DNA (cDNA) and amplified. Sequencing libraries suitable for the Illumina sequencing platform were constructed after partial cDNA fragments and splicing. The remaining cDNA was enriched for TCR, and the enriched products were amplified by PCR to construct a sequencing library suitable for the Illumina sequencing platform. Finally, each library was sequenced on an Illumina HiSeq X platform with 150 bp paired-end reads.

### Cell–cell interaction analysis

Cell–cell interactions (CCIs) between the eight cell types were predicted by Cellphone DB version [34] based on known ligand–receptor pairs. The permutation number was set to 1000 to calculate the null distribution of average ligand–receptor pair expression in randomized cell identities. The threshold cut-off of individual ligand or receptor expression was based on the average log of the gene expression distribution for all genes of each cell type. Predicted interaction pairs were visualized by the circlize (0.4.10) R package, and a p value < 0.05 and average log expression > 0.1 were considered significant pairs.

### Kaplan–Meier-plotter database analysis

Kaplan–Meier-plotter database (http://kmplot.com/analysis/) was applied to compare the overall survival of TNBC patients with different mRNA expression levels of *IGKC*, *IGHG1*, *IGHG4*, *SCGB2A1*, *PTN* and *MUCL1*. The population included in the analysis were patients with TNBC, and the detailed datasets of the included population are shown in Additional file 11: Table S4. The cutoff value of each molecule expression was determined by the median expression level in the population.

### GEO datasets analysis

TNBC datasets (GSE76124, GSE95700, GSE103091, GSE135565, GSE157284 and GSE167213) from the GEO database (https://www.ncbi.nlm.nih.gov/geo/) were applied to explore the associations of the expressions of *ERBB2*, immunoglobulin-related molecules (*IGHG1*, *IGHG4*, *IGKC* and *IGLC2*), and immunotherapy related targets (*PDCD1*, *CD274*, *CD47*, *CTLA4*, *CDK6* and *DDR2)*. The R package “sva” was applied to normalize the expression in different batches after merging the six GEO datasets. The correlation between expressions of immunoglobulin-related genes and the functions of immune microenvironment was also explored using Spearman test.

### IHC, FISH and immunofluorescence

For immunohistochemical analysis of ER, PR and HER2, the tissue paraffin blocks from seven TNBC puncture specimens were sectioned for analysis of ER, PR, and HER2 via IHC. Briefly, a 4-μm-thick tissue was deparaffinized, rehydrated and blocked by peroxidase after antigen retrieval, and a primary antibody (ER, Roche Diagnostics GmbH, Germany; PR, Roche Diagnostics GmbH, Germany; HER2, Roche Diagnostics GmbH, Germany) was incubated at room temperature for 3 h. Then, the slides were incubated with corresponding secondary antibody at 37 °C for 30 min, rinsed with PBS and stained with DAB substrate. Subsequently, routine dehydration, transparency, drying and sealing of tablets were conducted. Finally, the stained tablets were observed under a microscope (Olympus IX73), and images were evaluated independently by two experienced pathologists according to ASCO/CAP guidelines [18, 19]. In this study, only P2 was HER2 IHC 2 + , so FISH detection of HER2 was further conducted.

For fluorescence in situ hybridization (FISH) of HER2, the sections were detected using the HER2 FISH detection kit (Beijing Jinbojia Biotechnology Co., Ltd., China) following the manufacturer’s instruction. In brief, sections were routinely dewaxed in xylene, rehydrated with graded ethanol, treated with acidic sodium sulfite, digested with protease, soaked in 1% HCl, dehydrated with graded ethanol, fixed in acetone, baked at 56 °C for 5 min, added with probe working solution on the tissue sections, and denatured at 73 °C for 5 min. After that, the hybridization was performed overnight at 42 °C in a wet box for 16 h. Then, sections were rinsed with 50% formamide, citrate buffer, 0.1% NP-40 and 70% ethanol. Subsequently, the sections were dried naturally and counterstained with DAPI stain. After placing in the dark for 20 min, the sections were observed under a fluorescence microscope (Olympus IX73). The results were evaluated according to ASCO/CAP guidelines [18, 19].

For immunofluorescence (IF) analysis of IGHG4, IGKC, APOD, and MUCL1, before the antibody incubation, 4-μm-thick clinical paraffin section samples were deparaffinized and rehydrated, and the antigens were repaired through the microwave heating method. Then, tissue sections were blocked with 10% goat serum for 1 h in room temperature. After incubation of the primary antibody (IGHG4, 1:100, Proteintech, 66,408–1-Ig; IGKC, 1:200, Bioss Antibodies, Bs-3800R; APOD, 1:100, AB Clonal Technology, A15639; MUCL1, 1:200, Bioss Antibodies, Bs-17247R) at 4 times. Image quantification and analysis of each sample was done using Image J software. The staining intensity of each sample was the average staining intensity of 5 non-overlapping representative fields (× 200).

### Statistics

Descriptive statistics were used to delineate the clinicopathological characteristics of the retrospective study population. Continuous variables were presented as median and interquartile range and were compared using Wilcoxon rank-sum test. Categorical variables were expressed as counts and percentages and compared using the Fisher’s exact test. We used Kaplan–Meier and logrank test to compare the overall survival of HER2_low_ and HER2_neg_ TNBC patients diagnosed in our institution during the study period. We applied the Cox proportional hazards model to determine independent clinical risk factors for overall survival. In the prospective study of scRNA-seq, statistical analyses were performed with R software (version 3.6.1). Comparisons of the mean proportions of the eight cell types between the two TNBC groups were calculated using Student’s t test. Student’s t test was also used to quantitatively analyze the staining intensity of IGKC, IGHG4, APOD and MUCL1 in breast tumor tissues (HER2_low_ TNBC, n = 5, HER2_neg_ TNBC, n = 5) of the two groups. Logrank test was also applied to compare the overall survival of TNBC patients with different mRNA expression levels of *IGKC*, *IGHG1*, *IGHG4*, *SCGB2A1*, *PTN* and *MUCL1*. All the correlation analyses were performed by Spearman test. The expression levels of key immunotherapeutic biomarkers in the two TNBC groups were used Wilcoxon’s rank-sum test and corrected for multiple testing using Bonferroni’s test. *P* < 0.05 indicates statistical significance in this study.

## Results

### Patients with HER2_low_ TNBC show more malignant clinical features compared with HER2_neg_ phenotype

A total of 284 consecutive patients diagnosed with TNBC based on preoperative pathology were enrolled in this study. Finally, 251 patients met the inclusion criteria and entered in the further analysis, including 157 HER2_low_ (62.5%) and 94 HER2_neg_ TNBC patients (37.5%) (Fig. 1A). The clinicopathological characteristics and prognosis of the two groups were investigated. It showed that compared with HER2_neg_ TNBC patients, HER2_low_ patients were more prone to have larger tumor size (*P* = 0.04), lymph node involvement (*P* = 0.02), higher status of Ki67 (*P* < 0.001) and higher histological grade of lesions (*P* < 0.01); however, HER2_neg_ TNBC patients were more likely to be diagnosed at a younger age (< 45 *vs.* ≥ 45 years, *P* = 0.03, OR (95% CI) = 1.97 (1.09–3.57); Table 1). Notably, the Kaplan–Meier analysis revealed that HER2_low_ TNBC patients had a shorter overall survival (*P* < 0.001, HR (CI 95%) = 3.44 (2.10–5.62); Fig. 1B). Moreover, the Cox proportional hazards analysis showed that receiving the neoadjuvant systemic therapy, lymph node metastasis, Ki67 level ≥ 30%, tumor size > 2 cm and the higher histological grade (grade III) were significantly associated with the inferior prognosis of TNBC patients (Fig. 1C). Stratified analysis showed that the first three clinical characteristics above were significantly associated with the prognosis in HER2_low_ TNBC patients; however, these results were not observed in the HER2_neg_ group (Fig. 1C). Taken together, these clinical data indicated that TNBC patients with different HER2 phenotypes show distinct clinical features; HER2_low_ TNBC patients exhibit more malignant clinical behavior.

**Table 1:** The clinical characteristics of HER2_neg_ and HER2_low_ TNBC patients

Clinical characteristics	TNBC grouping (N = 251)		OR	95% CI	*P*
	HER2_neg_	HER2_low_			
	n = 94 (37.5%)	n = 157 (62.5%)			
Age (years)			1.97	1.09–3.57	**0.03**
< 45	29 (30.9)	29 (18.5)			
≥ 45	65 (69.1)	128 (81.5)			
Menstrual status			0.68	0.39–1.17	0.17
Pre	59 (62.8%)	112 (71.3%)			
Post	35 (37.2%)	45 (28.7%)			
Lesion			0.95	0.57–1.58	0.90
Left	46 (48.9%)	79 (50.3%)			
Others	48 (51.1%)	78 (49.7%)			
NST			0.91	0.54–1.51	0.80
No	48 (51.1%)	84 (53.5%)			
Yes	46 (48.9%)	73 (46.5%)			
Tumor size			1.93	1.04–3.57	**0.04**
< 2 cm	26 (27.7%)	26 (16.6%)			
≥ 2 cm	68 (72.3%)	131 (83.4%)			
Lymph node metastasis			1.85	1.10–3.13	**0.02**
Negative	45 (47.9%)	52 (33.1%)			
Positive	49 (52.1%)	105 (66.9%)			
Distant metastasis			0.85	0.31–2.30	0.8
Negative	87 (92.6%)	147 (93.6%)			
Positive	7 (7.4%)	10 (6.4%)			
Pathological type			1.53	0.85–2.77	0.16
Ductal	73 (77.7%)	109 (69.4%)			
Others	21 (22.3%)	48 (35.5%)			
Ki67, %			5.97	3.16–11.31	**< 0.001**
< 30	41 (43.6%)	18 (11.5%)			
≥ 30	53 (56.4%)	139 (88.5%)			
Histological grade			2.68	1.41–5.12	**< 0.01**
I/II	79 (84.0%)	104 (66.2%)			
III	15 (16.0%)	53 (33.8%)			

NST, neoadjuvant systemic therapy

*P* values in bold indicate statistical significance

### Tumor cell clusters in HER2_low_ TNBC have more aggressive signatures than HER2_neg_ TNBC revealed by scRNA-seq

**Fig. 2:**
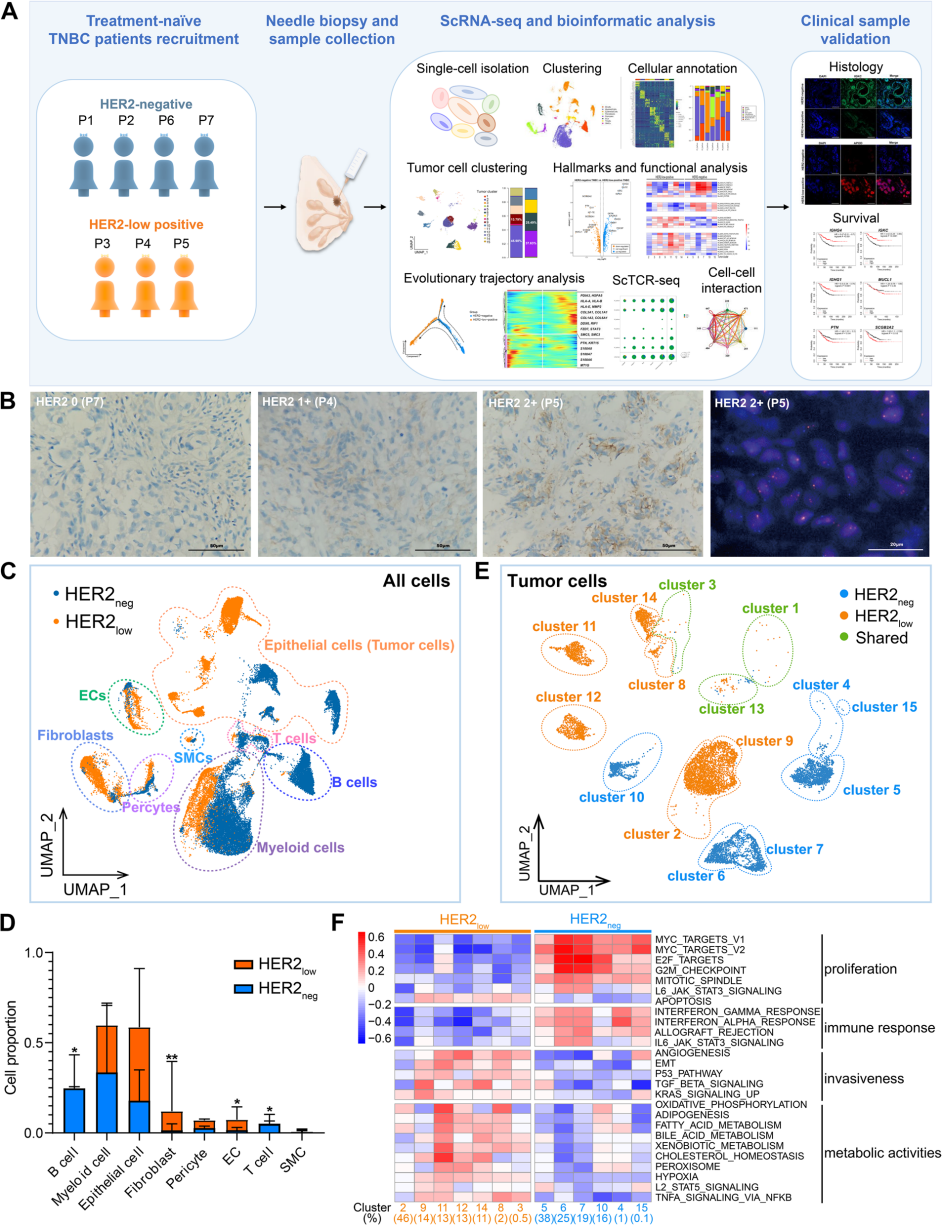
Different subclusters and signatures of tumor cells between HER2_low_ TNBC and HER2_neg_ TNBC. (**A**) Graphic view of the study design. (**B**) Representative IHC and FISH images showing different HER2 status (IHC 0, 1 + , 2 +) in tumor tissues from three primary TNBC patients (P7, P4 and P5). Scale bars: IHC, 50 μm; FISH, 20 μm. (**C**) UMAP plot of all the single cells in seven TNBC samples, containing eight identified cell types. SMCs, smooth muscle cells; ECs, endothelial cells. (**D**) Comparisons of the mean proportions of the eight cell types between the two TNBC groups. *P* value, Student’s t test (*P < 0.05; **P < 0.01). EC, endothelial cell; SMC, smooth muscle cell. See Additional file 13: Table S6 for details. (**E**) UMAP plot of tumor cells of HER2_low_ TNBC and HER2_neg_ TNBC. Orange, HER2_low_ TNBC; blue, HER2_neg_ TNBC; green, the common tumor clusters shared in two groups. (**F**) Heatmap of pathway activation scores by GSVA in two TNBC groups of the unique tumor cell clusters. Shown are GSVA scores from a lineal model. Tumor cell clusters are indicated on the top. The scores were estimated using SCENIC analysis with a Wilcoxon rank-sum test

High-quality data of 36,168 single cells totally were obtained by scRNA-seq from seven initial TNBC samples (HER2_low_, n = 3 *vs.* HER2_neg_, n = 4; Fig. 2A, B; Additional file 9: Table S2), including epithelial cells, stromal cells and immune cells according to their unique gene markers (Fig. 2C; Additional file 1: Fig. S1A; Additional file 10: Table S3). The cellular compositions were different between HER2_low_ TNBC and HER2_neg_ TNBC (Fig. 2D). Compared with HER2_low_ TNBC, HER2_neg_ patients had a relatively lower proportion of endothelial cells (ECs) (1.8% *vs.* 8.1%, *P* < 0.05) and fibroblasts (2.1% *vs.* 17.5%, *P* < 0.01) but more B cells (25.4%* vs.* 0.3%, *P* < 0.05) and T cells (6.1% *vs.* 0.7%, *P* < 0.05; Fig. 2D; Additional files 12, 13: Table S5, S6).

The tumor cells identified by CNV (Additional file 1: Fig. S1B) exhibited substantial heterogeneity among patients according to intratumoral heterogeneity (ITH) scoring and the UMAPs based on different patients (Additional file 1: Fig. S1C; Additional file 2: Fig. S2A). Fifteen subclusters of tumor cells were further subdivided based on their characteristic gene expression profiles which showed distinct clusters distribution between HER2_low_ and HER2_neg_ TNBC (Fig. 2E; Additional file 1: Fig. S1D). Although patients within the same TNBC group had different tumor subclusters, these subclusters exhibited similar functional characteristics (Fig. 2F; Additional file 2: Fig. S2B). Specifically, apart from the commonly shared cluster 1, 3 and 13, the HER2_low_ group (P3, P4 and P5) contained seven unique tumor subclusters, including clusters 2, 3, 8, 9, 11, 12 and 14, which highly expressed hallmarks of angiogenesis, EMT, and biological metabolic processing (Fig. 2F; Additional file 2: Fig. S2B), suggesting the aggressive signature of tumor cells in this group. By contrast, the HER2_neg_ TNBC group (P1, P2, P6 and P7) was consisted of signature tumor clusters 4, 5, 6, 7, 10, and 15, which were associated with cell proliferation and immune response process (Fig. 2F; Additional file 2: Fig. S2B); particularly, the major clusters (cluster 5 and 6) were responsible for antigen processing and presentation. Altogether, these findings suggest HER2_low_ and HER2_neg_ TNBC have heterogeneity in tumor cluster subdivisions and characteristic functional signatures; HER2_low_ TNBC have more aggressive tumor cell clusters than HER2_neg_ TNBC.

### HER2_low_ TNBC and HER2_neg_ TNBC have different tumor evolutionary characteristics

**Fig. 3:**
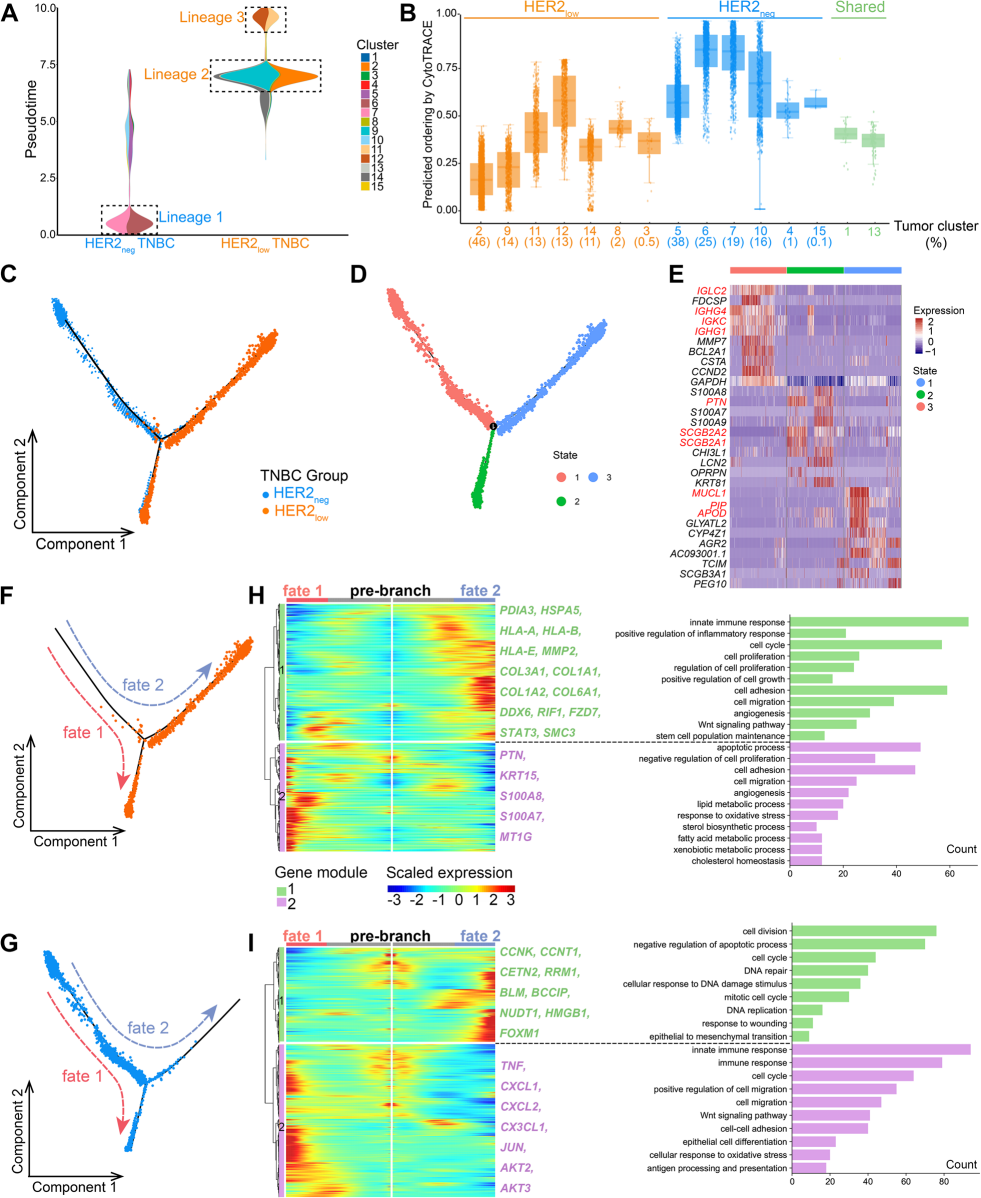
Different dynamic evolutionary characteristics of tumor cells in HER2_low_ and HER2_neg_ TNBC. (**A**) Density of tumor cells along pseudotime in two TNBC groups. The vertical axis is the pseudotime process from bottom to top, the horizontal axis is the composition of cell clusters at different pseudotime state (displayed by TNBC groups). The main tumor clusters of the two groups have been annotated, diverging from early to late lineages 1,2, and 3, respectively. **(B**) The box plot shows the differentiation status of each tumor cell subcluster using CytoTRACE. Tumor clusters marked in blue and orange fonts are specific clusters of HER2_neg_ and HER2_low_ TNBC, respectively, and the clusters marked in green font are shared by the two TNBC groups. (**C**) Trajectory of the evolution of tumor cells predicted by monocle. The dashed arrows indicate the direction of evolution. (**D**) Three states of tumor evolution in all tumor cells. From State 1 to State 3 indicates that tumor cell evolution from early to late stages. (**E**) Heatmap indicates the gene expression signatures of the three evolutionary states. (**F**) Trajectory reconstructions of tumor cells in the HER2_low_ TNBC and (**G**) HER2_neg_ TNBC, respectively, revealing three branches including pre-branch, fate 1, and fate 2. (**H**) Heatmaps show upregulated or downregulated genes along with the two differentiation fates and the GO enrichment analysis in HER2_low_ and (**I**) HER2_neg_ TNBC groups, indicating active signature pathways for each branch in two groups. The abscissa is from the middle to the left and right sides (fate 1, fate 2), representing the tumor evolution process from early to late; the ordinate represents the gene, and each point represents the average expression of the specified gene in the pseudotime. Genes with similar expression patterns are clustered into one module

We found tumor clusters in the two TNBC groups had different differentiative status. The clusters in HER2_low_ TNBC were at a higher differentiative state but maintained a lower tumor stemness level (Fig. 3A, B). However, main clusters in HER2_neg_ TNBC presented the earliest state of differentiation (lineage 1; Fig. 3A) which showed the upregulation of immune activation (such as GO term “antigen processing and presentation”; Additional file 3: Fig. S3A). Furthermore, the HER2_low_ TNBC group exhibited two cell lineages with different differentiation states: one lineage included clusters 2, 9, and 14 (lineage 2; Fig. 3A) at a moderate differentiation stage; the other lineage (lineage 3) containing clusters 11 and 12 was in the latest stage (Fig. 3A), playing a part in metabolism-related activities (Additional file 3: Fig. S3A). Moreover, our further analysis showed that the higher expression level of *ERBB2*, the higher differentiation stage of tumor cells, indicating a possible connection between *ERBB2* expression and tumor development in TNBC (Additional file 3: Fig. S3B).

Then, the developmental and evolutionary characteristics of the two groups were further investigated. Interestingly, the pseudotime trajectory almost began with HER2_neg_ tumor cells and then split into two divergent differentiated branches (Fig. 3C). One terminal end of the trajectory was the mixture of HER2_neg_ and a part of HER2_low_ tumor cells; in contrast, the other terminal end was full of HER2_low_ tumor cells (Fig. 3C). Then, all tumor cells were divided into three states according to the pseudotime trajectory (Fig. 3D). Intriguingly, tumor in state 1 exhibited an activation of immune-related signatures (*IGLC2*, *IGHG4*, *IGHG1* and *IGKC*; Fig. 3E, Additional file 4: Fig. S4), while the tumor cells in state 3 which were mostly contributed by HER2_low_ cells possessed the characteristics of metabolic biological function with the highly expression of *PTN*, *SCGB2A2*, *MUCL1*, *PIP*, etc. (Fig. 3E, Additional file 4: Fig. S4). Of note, some of the genes (*PIP* and *APOD*) are well-characterized androgen receptor (AR) target genes and the pathway analysis was enriched in lipid, fatty acid and cholesterol pathways that typically are elevated in luminal AR TNBC subtypes. Thus, we further evaluated *AR* and other AR target genes (*FKBP5*, *PIP*, *APOD*, *ALCAM*, *DHCR24*, *FASN*, *CLDN8*) in this trajectory to explore whether *AR* could contribute to the higher differentiation. Interestingly, our data appeared that almost all of these genes (except *FKBP5*) had similar expression patterns, with expression increasing gradually along the pseudotime trajectory (Additional file 5: Fig. S5A), especially in HER2-low TNBC (Fig. 3C; Additional file 5: Fig. S5B). Therefore, it would be worth staining HER2-low for AR and quantifying in TNBC patients.

The two differentiation branches (fate 1 and fate 2) of the two groups were, respectively, presented (Fig. 3F, G). In HER2_low_ TNBC group, the fate 1 branch was predominantly associated with module 2 genes (*PTN*, *KRT15*, *S100A8*, etc.) which suggested the tumor harbored the features of apoptosis, migration and metabolism (Fig. 3H). The fate 2 branch presented higher levels of module 1 genes enriched in immune and inflammatory process (such as *PDIA3*, *HSPA5* and *HLA-A*), cell proliferation and migration (e.g., *MMP2*, *COL3A1* and *COL1A1*), as well as cell stemness maintenance (e.g., *DDX6*, *RIF1* and *SMC3*) (Fig. 3H). Likewise, in HER2_neg_ TNBC group, two gene modules were observed during the two differentiated fates. Module 1 genes were mainly involved in cell proliferation (*CCNK*, *CCNT1*, *CETN2*, etc.) and DNA damage repair (*BLM*, *HMGB1*, *FOXM*, etc.), which were highly expressed during fate 2, whereas the module 2 genes, mainly elevated during fate 1, were enriched in immune response and tumor migration process, highly expressing *TNF*, *CXCL1*, *JUN*, etc. (Fig. 3I).

In summary, HER2_neg_ and HER2_low_ TNBC harbored distinct tumor clusters with distinct signatures as well as different evolutionary characteristics, indicating the biological heterogeneity of TNBC. To some degree, this is a probable cause of the clinical heterogeneity in TNBC patients with different HER2 status.

### HER2_neg_ TNBC exhibits higher expressions of immunoglobulin-related genes linked with a favorable prognosis than HER2_low_ TNBC

**Fig. 4:**
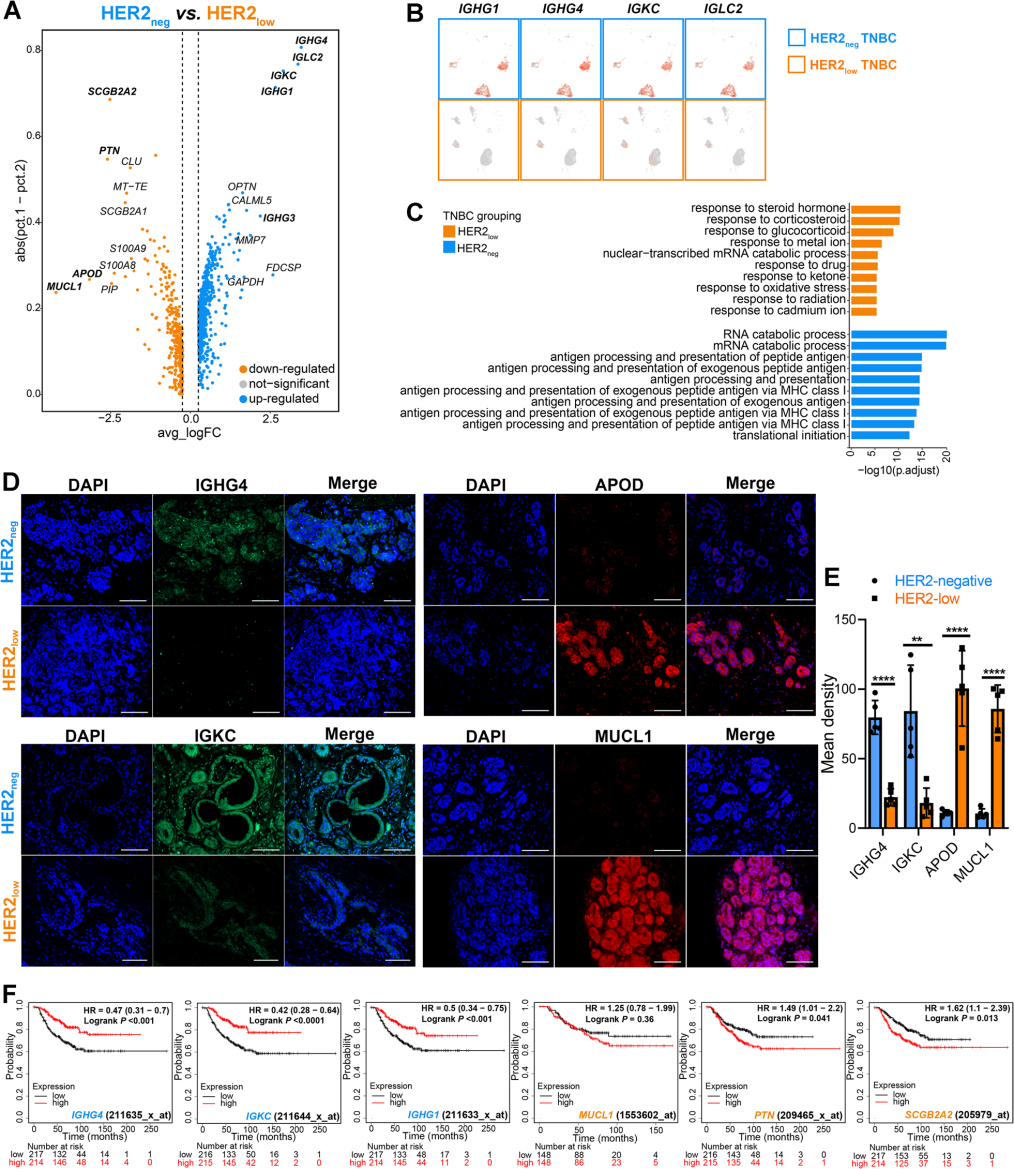
Different hallmarks of tumor between HER2_low_ TNBC and HER2_neg_ TNBC. (**A**) Volcano map shows the DEGs between two TNBC groups of tumor cells. Each point represents a gene; genes marked in orange and blue are highly expressed genes in tumor cells of HER2_low_ TNBC and HER2_neg_ TNBC, respectively. (**B**) The expression levels of four representative immunoglobulin-related genes in tumor cells of HER2_neg_ and HER2_low_ TNBC, respectively. (**C**) GO (BP) enrichment pathway analysis of the highly expressed DEGs of tumor cells in the two TNBC groups. (**D**) Representative images of fluorescent staining for the verification of IGHG4, IGKC, APOD and MUCL1 expressions in breast tumor tissues of two groups of clinical TNBC samples. All scale bars, 50 μm. (**E**) Quantitative analysis of the intensity of staining of IGHG4, IGKC, APOD and MUCL1 expressed in two groups of breast tumor tissues (HER2_low_ TNBC, n = 5) *vs.* HER2_neg_ TNBC, n = 5) by fluorescent staining. *P* value, Student’s t test (∗*P* < 0.05, ∗*P* < 0.01, ∗∗*P* < 0.001, ∗∗∗*P* < 0.0001). Error bars show SEM of single patients. (**F**) The overall survival of TNBC patients with different mRNA expression levels of *IGHG4*, *IGKC, IGHG1*, *SCGB2A1*, *PTN* and *MUCL1* using Kaplan–Meier-plotter database. The cutoff values were set as the median expression values of all above genes, and *P* < 0.05 indicates statistical significance, using logrank test

To further explore the underlying differences in tumor biological hallmarks between the two TNBC groups, the DEGs of the tumor cells were further investigated (Additional file 6: Fig. S6A). Notably, the immunoglobulin-related genes (*IGKC*, *IGHG1, IGHG4*, *IGLC2*, etc.) were significantly upregulated in HER2_neg_ TNBC, while *APOD*, *MUCL1*, *SCGB2A1*, *PTN*, etc., were upregulated in HER2_low_ TNBC (Fig. 4A, B). Of note, GO analysis showed the immune activation function was upregulated in HER2_neg_ TNBC, such as antigen processing and presentation, immune response-activating signal transduction and regulation of innate immune response (Fig. 4C). In addition, GSEA revealed that HER2_neg_ TNBC tumor was mediating some processes involving in immune-related activities, such as IL-6-JAK-STAT3 signaling (Additional file 6: Fig. S6B).

Then, we verified the expressions of IGHG4, IGKC, APOD and MUCL1 at the protein level in tumor sections of clinical TNBC patients (HER2_low_, n = 5 *vs.* HER2_neg_, n = 5) by IF staining. Strikingly, it showed that IGHG4 and IGKC were predominantly expressed in HER2_neg_ TNBC group (in tumor tissues containing tumor cells and mesenchymal cells), whereas APOD and MUCL1 were highly expressed in HER2_low_ group (Fig. 4D, E), which were consistent with the scRNA-seq data.

Furthermore, the clinical cohort in Kaplan–Meier-plotter database demonstrated that the higher expression of *IGKC*, *IGHG1* and *IGHG4* (which were highly expressed in HER2_neg_ TNBC) were significantly associated with favorable overall survival in TNBC patients; however, the high expressions of *SCGB2A1* and *PTN* (highly expressed in HER2_low_ TNBC) were linked to worse outcomes in TNBC patients (Fig. 4F).

In summary, these findings suggested that HER2_neg_ TNBC harbored distinct tumor properties from HER2_low_ TNBC phenotype which exhibited more common expressions of immunoglobulin-related hallmarks.

### HER2_neg_ TNBC reveals a potentially more active immune microenvironment than HER2_low_ TNBC

**Fig. 5:**
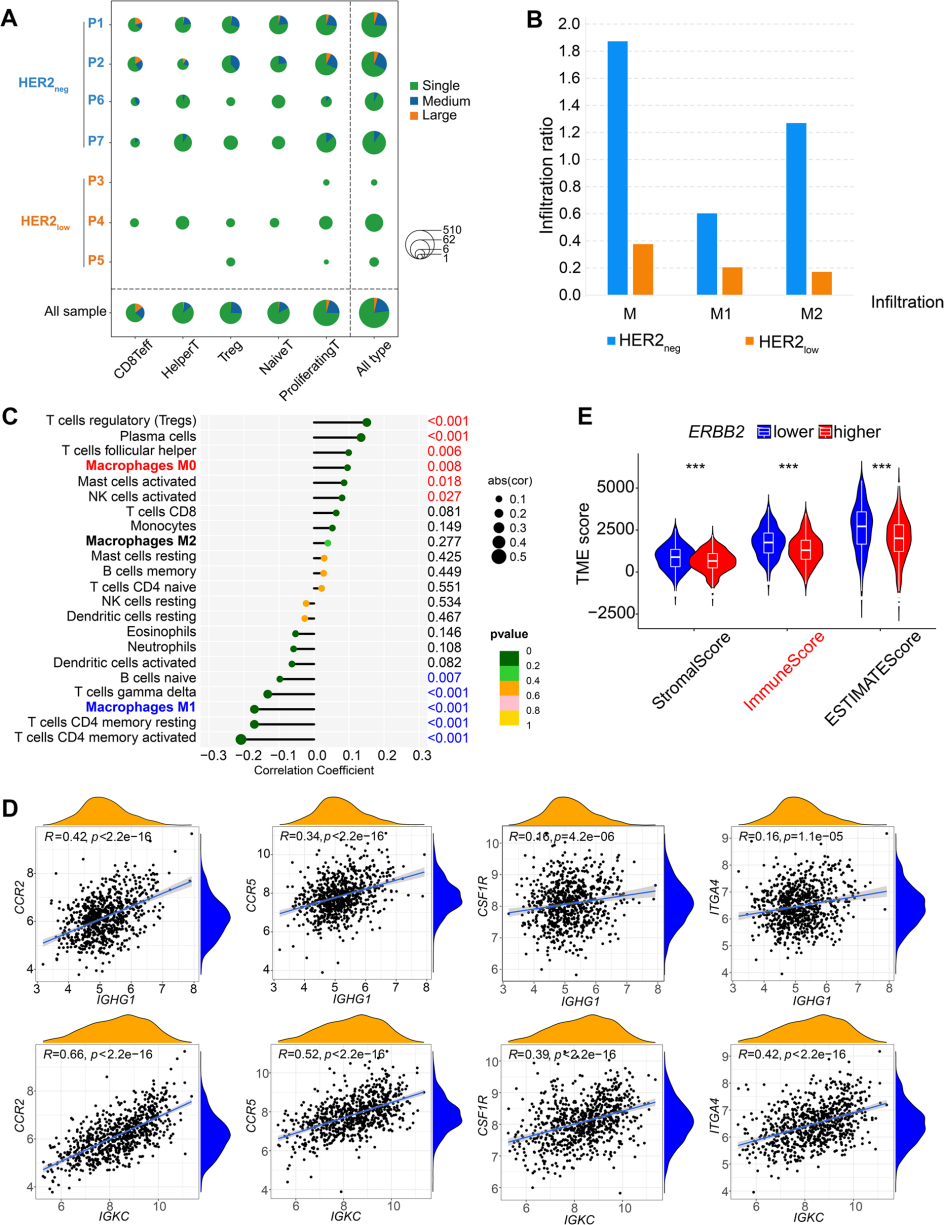
Distinct patterns of immune cell characteristics between HER2_low_ TNBC and HER2_neg_ TNBC. (**A**) scTCR-seq analysis shows TCR diversity in HER2_neg_ TNBC and HER2_low_ TNBC. The horizontal axis lists T cell types, and the vertical axis shows individual samples and TNBC groupings. Different colors represent different frequencies of TCR clonotypes. Single, unique TCR clonotypes; medium, TCR clonotypes with a frequency between 1 and 10; large, TCR clonotypes with a frequency > 10.The size of the circle represents the number of T cells. (**B**) Bar plot shows infiltration levels of different macrophages in HER2_neg_ and HER2_low_ TNBC groups. M, all macrophages; M1, M1 macrophages; M2, M2 macrophages. (**C**) Correlations between *ERBB2* mRNA expression level and various immune cell functions in TNBC (GSE76124, GSE95700, GSE103091, GSE135565, GSE157284 and GSE167213). *P*, Spearman correlation analysis; the numbers on the right are p values; p values in red indicate a significant positive correlation, while those in blue indicate a significant negative correlation. (**D**) Correlations between the expressions of characteristic immunoglobulin genes (*IGHG1*, *IGKC*) and the signature molecules in macrophage recruitment (*CCR2*, *CCR5*, *CSF1R*, *ITGA4*), using Spearman correlation analysis; *P* < 0.05 indicates statistical significance. (**E**) Comparison of immune scores in TNBC samples with different *ERBB2* expression levels. The median expression level of *ERBB2* was used as the cut-off value

The two groups of TNBC showed different diversity of immune cells (Additional file 7: Fig. S7A). Specifically, CD8^+^ effector T cells, CD4^+^ proliferating T cells and naïve T cells seemed more common in HER2_neg_ group. Single-cell T-cell receptor (TCR)-sequencing (scTCR-seq) showed that the HER2_neg_ group had more enriched TCR diversity with abundant clonotypes of TCR (Fig. 5A). Importantly, HER2_neg_ TNBC presented significantly higher macrophage infiltration (mainly M2 phenotypes) compared with HER2_low_ group (Fig. 5B). Meanwhile, the additional GEO datasets showed that the expression of *ERBB2* in TNBC was significantly positively correlated with M0 macrophages infiltration but negatively associated with the M1 macrophages infiltration which play an important role in in anti-tumor immune activities (*P* < 0.001; Fig. 5C). Furthermore, RNA-seq of TNBC cases revealed that *IGHG1* and *IGKC* (which were highly expressed in the HER2_neg_ TNBC) were both positively associated with *CCR2*, *CCR5*, *CSF1R* and *ITGA4* (Fig. 5D) which have been explained as crucial markers of the recruitment of macrophages [35]. In addition, the TNBC dataset revealed that the lower level of *ERBB2*, the higher immune and TME scores (Fig. 5E). Moreover, the communication between tumor and immune cells was more widespread in the HER2_neg_ group, especially in immune response-related crosstalk between tumor cells and myeloid cells (which was primarily comprised of macrophages; Additional file 7: Fig. S7B), such as “HLA-DPB1–TNFSF13B” and “TNFRSF1A–GRN” (Additional file 7: Fig. S7C). By contrast, HER2_low_ TNBC appeared to lack interactions in the tumor microenvironment with scarce crosstalk between tumor cells and immune cells (Additional file 7: Fig. S7C). Collectively, these findings together indicated that TNBC with distinct HER2 phenotypes have different immune states of the tumor microenvironment; HER2_neg_ TNBC reveals a more active state of immune microenvironment which is crucial for promoting the response of immunotherapies (Additional file 8).

### HER2_neg_ TNBC exhibits preferable response with a pro-inflammatory state of immune microenvironment after immunotherapy

**Fig. 6:**
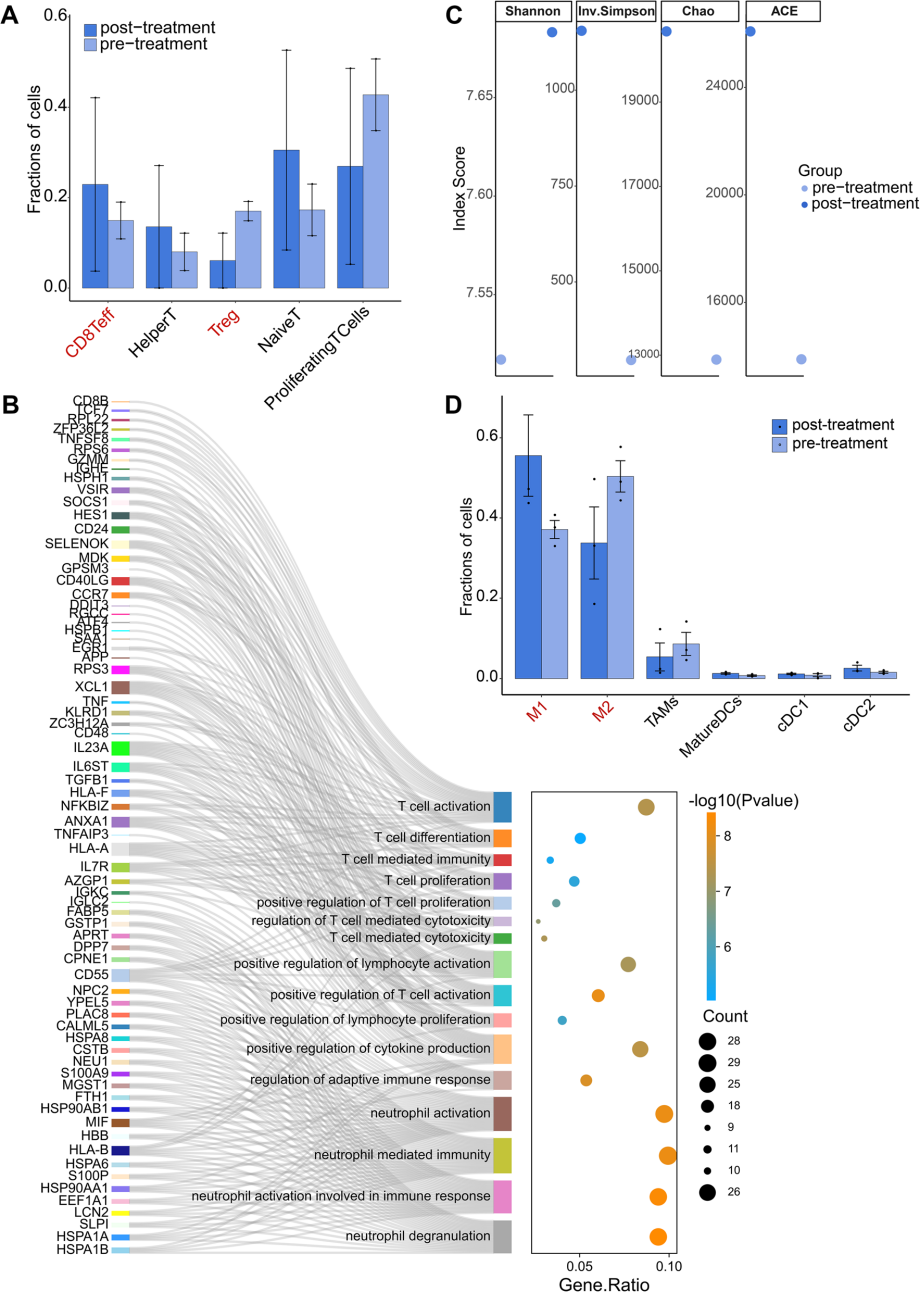
Characteristic changes of immune microenvironment in HER2_neg_ TNBC patients before and after neoadjuvant immunotherapy. (**A**) Comparison of the abundance of the subclusters of T cells before and after treatment of HER2_neg_ TNBC. CD8Teff, CD8^+^ effector T cells; HelperT, CD4^+^ helper T cells. (**B**) Pathway enrichment analysis of up-regulated genes in T cells after neoadjuvant immunotherapy in HER2_neg_ TNBC compared with treatment-naïve T cells. (**C**) ScTCR-seq analysis in HER2_neg_ TNBC before and after treatment by four different scoring methods. (**D**) Comparison of the abundance of the subclusters of microphages before and after treatment in HER2_neg_ TNBC patients. M1, M1 microphages; M2, M2 microphages; TAMs, tumor-associated microphages; MatureDCs, Mature dendritic cells; cDC, classical dendritic cells

After treatment, HER2_neg_ TNBC presented preferable clinical response (Additional file 9: Table S2) with an enhanced antitumor capacity of immune microenvironment. Compared with the treatment-naïve patients, patients after immunotherapy had decreased levels of Tregs, accompanied by elevated levels of CD8^+^ T cells and CD4^+^helper T cells (Fig. 6A). These augment of potential tumor-reactive T cells implicated an enhanced antitumor capacity of T cells after immunotherapy in HER2_neg_ TNBC patients, which was also manifested by both the upregulated functions of T cell involving in immune response after immunotherapy (Fig. 6B) and the exhibition of the higher TCR diversity than before (Fig. 6C) (Additional files 10, 11).

Of note, the abundance of M1 macrophages was increased, but M2 macrophages were decreased after NST (Fig. 6D), suggesting that NST may alter the degree of M1/M2 macrophage polarization in HER2_neg_ TNBC, which is conducive to M1 macrophage polarization but detrimental to M2 macrophage polarization, exerting as an important role in anti-tumor functions.

Altogether, our results suggests that the immune microenvironment may be more prone to a proinflammatory state after immunotherapy in HER2_neg_ TNBC patients, which potentially contribute to the response to immunotherapies.

### HER2_neg_ TNBC exhibits higher levels of immunotherapeutic biomarkers than HER2_low_ TNBC

**Fig. 7:**
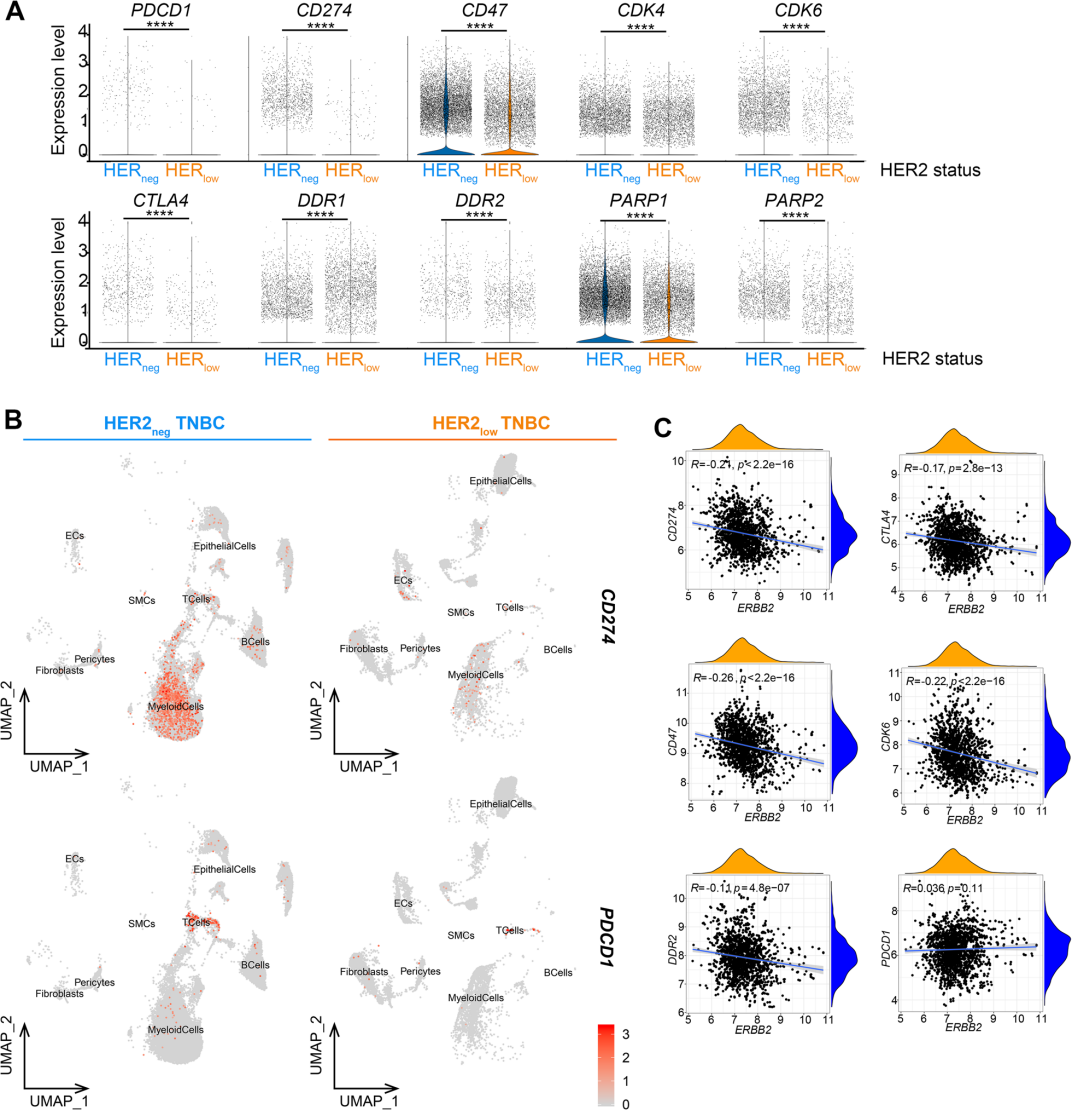
Different expression patterns of critical immunotherapeutic biomarkers in HER2_low_ TNBC and HER2_neg_ TNBC. (**A**) Violin plot shows the expression levels of key immunotherapeutic biomarkers in the two TNBC groups. See Additional file 14: Table S7 for details. (**B**) Color-coded UMAPs for expression levels (gray to red) of *PDCD1* and *CD274* in two TNBC groups. (**C**) Correlations between ERBB2 expression and immunotherapeutic targets (*CD274*, *CTLA4*, *CD47*, *CDK6*, *DDR2*, *PDCD1*), using Spearman correlation analysis; *P* < 0.05 indicates statistical significance

Interestingly, the tumor cells in two TNBC groups displayed different expression patterns of the critical immunotherapeutic targets, such as *PD-1/L1*, *CTLA4*, *CD47*, *CDK4/6*, *PARP1/2* and *DDR1/2* (Fig. 7A; Additional file 14: Table S7). Of note, *PDCD1* (PD-1) and *CD274* (PD-L1) were highly expressed on T cells and myeloid cells in the HER2_neg_ TNBC group but barely in the HER2_low_ group (Fig. 7B). It has been reported that the remarkable expression of PD-L1 on myeloid cells in host would also increase the potential risk of tumor immune escape [36]. Thus, the application of immunotherapy seems more imperative for HER2-negative TNBC patients. Meanwhile, the TNBC dataset from GEO database confirmed that *ERBB2* was negatively correlated with the expressions of *CD274*, *CTLA4*, *CD47, CDK6* and *DDR2*. Furthermore, the expression of immunoglobulin-related molecules was significantly positively correlated with the expression of these immunotherapeutic targets (Fig. 7C).

Overall, the immune microenvironments were probably at different states between HER2_low_ and HER2_neg_ TNBC showing as the distinctions on immune cell abundance, TCR diversity, as well as expression levels of critical immunotherapeutic targets. Therefore, it may deserve further consideration on HER2 status when immunotherapy is incorporated in TNBC patients.

## Discussions

**Fig. 8:**
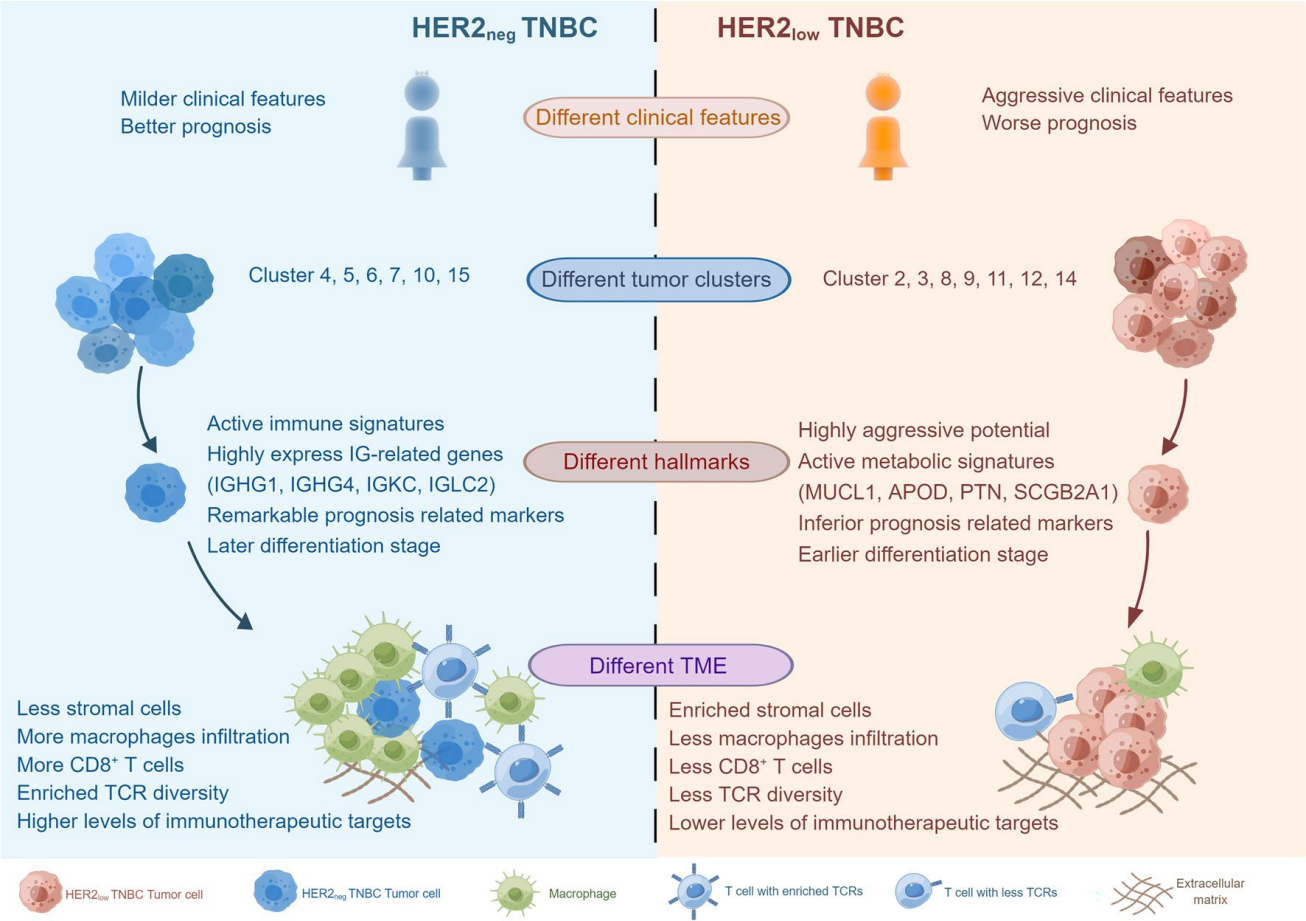
Clinical and biological heterogeneities in HER2_neg_ TNBC and HER2_low_ TNBC. HER2_low_ TNBC and HER2_neg_ TNBC show distinct clinical features as well as different tumor properties, including tumor cell clusters, tumor hallmarks, and TMEs

In this study, the potential clinical and biological heterogeneities of HER2_neg_ and HER2_low_ TNBC were explored by both the retrospective and prospective approaches. We found HER2_neg_ TNBC patients harbored milder clinical features than HER2_low_ phenotype with less lymph node involvement, lower histological grade of lesions, lower level of Ki67, and had a favorable outcome. Then, we further investigated the underlying differences of single-cell transcriptome profiling between HER2_low_ and HER2_neg_ TNBC (Fig. 8). Our data suggested these two TNBC phenotypes harbored distinct tumor properties with varying biological features. The HER2_low_ tumors exhibited aggressive signatures associated with increased capacities for metabolism, proliferation and differentiation; while the HER2_neg_ tumors highly expressed immunoglobulin-related genes and were more likely to play a role in immune activities. Additionally, tumors of these two distinct TNBC groups presented different evolutionary dynamic trajectories and hallmarks. Moreover, HER2_neg_ TNBC exhibited enriched expression of immunotherapy-targeted genes and enhanced immunological activity with substantial CD8^+^ T cells and TCR diversity and would also affect the biological functions of macrophages. To the best of our knowledge, this is the first study to unveil the distinct biological properties of tumor and immune microenvironments between HER2_low_ and HER2_neg_ TNBC at single-cell RNA resolution. Altogether, our data revealed TNBC with different HER2 status harbored different patterns of tumor features as well as immune microenvironment characteristics. This study highlights an important role of HER2 in the heterogeneity of TNBC tumorigenesis and may provide new insights into the development of more refined clinical classification and novel tailored therapies for TNBC patients.

TNBC is known to exhibit heterogenous characteristics, so the identification of its intron subtypes is imperative for understanding the underlying biological behavior and facilitating personalized treatment strategies. Extensive efforts have been devoted to expound various potential subgroups of TNBC on the basis of its unique molecular characteristics, such as VICC [5], Baylor [37] and FUSCC types [38]. Notably, the FUSCC typing subdivided TNBC into four distinct subgroups, and its further clinical study (FUTURE trial, NCT03805399) suggested that the combination of pyrotinib and capecitabine was effective even if the expression of HER2 was clinically negative for some patients [39]. Therefore, IHC-based classifications of TNBC may enable us to better evaluate the therapeutic benefit, but it is not yet known whether HER2-negativity could serve as a contraindication criterion for HER2-targeted therapy.

The signature of HER2 in BC has evolved dramatically over the past three decades, from a poor prognostic biomarker to one of the clinical targets for some anti-HER2 drugs, especially for patients with HER2-enriched tumors. The emergence of HER2-targeted drugs has improved the prognosis of BC patients with abnormal amplification or overexpression of *ERBB2* [40], but at present, this agent has not yet been approved for patients with HER2_low_ TNBC. Although patients with “low-expression of HER2” are diagnosed as “HER2_neg_” currently, different levels of HER2 are still expressed on the surface of tumor cells of TNBC [41], probably associated with tumor clinical features to some extent [42]. However, recent studies on the effect of HER2_low_-status on prognosis for TNBC patients were inconsistent. Some studies suggested that low-expression of HER2 did not affect the prognosis of TNBC [13, 17, 43], or it was linked to a better clinical outcome (compared with HER2_neg_ TNBC) [8]. However, the data involved in these studies were mostly based on patients’ chemotherapy results, and the results of patients who received targeted therapy or immunotherapy were not included. Of note, some immunotherapeutic targeted regimes have been regarded as promising candidates for TNBC treatment which can improve the survival of TNBC patients with the combination with chemotherapeutic agents [44–47]. Moreover, a large number of clinical trials of novel targeted drugs are underway in patients with HER2_low_ breast cancer [14]; the results are yet to be published to date, but some novel HER2-targeted agents (NCT02277717, NCT03734029) and antitumor vaccines (NCT01570036, NCT01570036) have shown promising activity in HER2_low_ TNBC patients. Therefore, although the subdivisions of TNBC according to HER2 status in the future remains to be verified, the research on the difference between HER2_low_ and HER2_neg_ TNBC is of great significance. Hence, our investigations in this study are pivotal for TNBC scenario.

Regarding the tumor characteristics, our data revealed TNBC with HER2_low_/HER2_neg_ expressions have different tumor cellular compositions as well as functional hallmarks. The discrepancy in both composition and functions of tumor clusters could be observed between HER2_low_ and HER2_neg_ TNBCs, and they harbored different differentiation states, which may give a hint on the diverse tumor identities between these two TNBC groups. Recent studies indicated that HER2 0 and HER2 1 + /2 + (by IHC, the same below) BC had completely different intrinsic subtype distributions in PAM50 [11] as well as varying genetic backgrounds [8]. Likewise, our data showed HER2_low_ TNBC actively participated in activities with regard to tumor metabolism and growth pathways with *MUCL1*, *PTN*, *SCGB2A2* and *APOD* highly expressed, revealing an increased metabolic and proliferative capacity contained. In addition, the two TNBC groups also presented as distinct tumor evolutionary dynamics and characteristics. Compared with HER2_neg_ TNBC, HER2_low_ tumor was at a relatively later stage of evolution with a higher level of differentiation. The different gene expression patterns within the two groups of TNBC manifested as distinct differentiation pathways and functional characteristics, potentially give a hint to further treatment optimizing for different TNBC individuals.

Importantly, tumor cells in HER2_neg_ TNBC seemed more likely to be associated with activities involving immune responses, highly expressing immunoglobulin-related genes (such as *IGKC*, *IGHG1, IGHG4* and *IGLC2*) which are associated with the favorable prognosis and have been verified in the clinical TNBC cases. Immunoglobulin is a class of globulin with antibody activity, which is an important component of the body to resist disease [48]. Traditionally, it is believed that only B lymphocytes and plasma cells can produce and secrete immunoglobulins [49, 50]. However, more and more studies have discovered that various tumor cells (e.g., breast, cervical, lung cancer) can also express immunoglobulins (especially IgG) which play an important role in the occurrence and development of cancer [51–56]. In this study, we found that tumor-derived immunoglobulins were expressed more common in HER2_neg_ TNBC than in HER2_low_ TNBC. In addition, the expression some immunoglobulins were positively correlated with the function of macrophage recruitment and expressions of critical therapeutic targets in TNBC, suggesting immunoglobulins may play a potential role in immune regulation processes. Therefore, further investigation of the implications of tumor-derived immunoglobulins in TNBC will be helpful to formulate new strategies for the refined diagnosis and treatment.

To explore the impact of HER2_low_ on immune activities in TNBC, we compared the difference in immune microenvironment between HER2_low_ and HER2_neg_ TNBC. Our results suggested the abundance in subdivisions of T cells, B cells and myeloid cells were different between these two groups; the expression of *ERBB2* was correlated with functions of various immune cells as well as TME scores, which might collectively contribute to a heterogeneity in immune activation between these two TNBC patient groups. Of note, we found HER2_neg_ TNBC presented higher M2 infiltration, which seems to contradict the common belief that M2 has a pro-tumor effect resulting in poor prognosis. Interestingly, although more M2 infiltration was estimated than M1 in the HER2_neg_ group, the infiltrations of M1 and M2 in the HER2-negative group were both higher than that in the HER2_low_ group. In addition, macrophage polarization is plastic and can switch with tumor progression via complicated regulatory mechanisms, and the activation of M1 or M2 is affected by many factors [57, 58]; thus, the role of macrophage polarization here still needs to be further explored. In light of the prominent role of key immune checkpoint genes, (such as PD-1/L1) in the immunosuppression of the immunosuppressant tumor microenvironment [59, 60], we also compared the expression patterns of these critical immune checkpoint genes between these two different TNBC groups. Intriguingly, both *PDCD1* (PD-1) and *CD274* (PD-L1) were highly expressed in HER2_neg_ TNBC instead of HER2_low_ group. Furthermore, we confirmed in a larger TNBC samples that *ERBB2* level was reversely related to expressions of these immune therapeutic biomarkers. Altogether, these data suggested that the heterogeneity of HER2 in TNBC patients may affect the efficacy of immunotherapy; HER2_neg_ TNBC would more likely to get a potential benefit from immunotherapy in the clinical management. Although it remains further explored, the HER2 heterogeneity may be a non-negligible factor when considering immunotherapy-based therapy for clinical treatment of TNBC patients in the future.

The major limitation of this study included the relatively small sample size of patient biopsy samples, which might partially compromise the statistical significance of our findings. Additionally, due to the lack of well-established in vitro cell culture models of HER2_low_ TNBC to date, further functional validations were hindered. To address this issue, the staining of additional clinical samples and further analyses with larger sample size based on public databases were conducted. At present, the clinical definition of “HER2-low” is only based on the histological level, and has not yet penetrated into the RNA level, so we used the median RNA expression level of *ERBB2* in the TNBC population as the cut-off value, which might be biased. Moreover, in the process of our clinical data statistics, we found that some patients’ puncture results and postoperative pathological test results were inconsistent, especially for the drift of HER2 IHC1 + and 2 + . Although the definition of IHC1 + and 2 + will not have any impact on the conclusions of this study, more stringent requirements for the selection of patients with IHC1 + and 2 + should be employed if more refined studies are to be conducted in the future. Therefore, improvements in more accurate and standardized detection of HER2 expression are still urgent for TNBC patients.

## Conclusions

Taken together, our data invoke a key issue that TNBC patients with different HER2 status harbor distinct clinical behavior and tumor biological properties. The heterogeneity of HER2 expression may be a non-negligible factor in the clinical management of TNBC patients. Our study has shed new light on the inherent heterogeneity of TNBC with different HER2 status, providing new clues to the development of a more refined classification and tailored therapeutic strategies for TNBC patients.

## Supplementary Information

**Additional file 1**: **Fig. S1**. Showing the single-cell landscape of all TNBC samples in this study.

**Additional file 2**: **Fig. S2**. Showing the UMAPs of tumor cells based on different patients and the expression of four key biological functions in different tumor clusters which suggests HER2_neg_ TNBC and HER2_low_ TNBC harbor distinct tumor biological properties.

**Additional file 3**: **Fig. S3**. Showing tumor cell subclusters with different differentiation status had distinct features and *ERBB2* levels.

**Additional file 4**: **Fig. S4**. Showing GO enrichment pathways of highly expressed genes of all tumor cells at three evolutionary states during the pseudotime.

**Additional file 5**: **Fig. S5**. Showing the expression characteristics of AR and AR target genes in the pseudotime trajectory.

**Additional file 6**: **Fig. S6**. Showing different hallmarks of tumor cells in HER2_low_ TNBC and HER2_neg_ TNBC.

**Additional file 7**: **Fig. S7**. Showing different immune microenvironments of HER2_low_ TNBC and HER2_neg_ TNBC.

**Additional file 8**: **Table S1**. Showing the detailed clinical information and follow-up data of the enrolled TNBC population.

**Additional file 9**: **Table S2**. Showing the clinical information of seven primary triple-negative breast cancer patients with different HER2 status.

**Additional file 10**: **Table S3**. Showing gene markers of all cell types in this study.

**Additional file 11**: **Table S4**. Showing TNBC population included for overall survival analysis in Kaplan–Meier database.

**Additional file 12**: **Table S5**. Showing cellular composition of the seven TNBC samples.

**Additional file 13**: **Table S6**. Showing the mean proportion of the eight main cell types in the two TNBC groups.

**Additional file 14**: **Table S7**. Showing the expression level of key immunotherapeutic biomarkers in HER2_neg_ TNBC and HER2_low_ TNBC.

## Data Availability

The scRNA-seq datasets supporting the conclusions of this article are available in the Genome Sequence Archive (GSA) database, under the accession number HRA002137 in https://ngdc.cncb.ac.cn/gsa-human. TNBC datasets (GSE76124, GSE95700, GSE103091, GSE135565, GSE157284 and GSE167213) can be accessed from the GEO database (https://www.ncbi.nlm.nih.gov/geo/). Other supporting data are included within the additional files or available from the corresponding author upon reasonable request.

## Abbreviations

BC: Breast cancer
TNBC: Triple-negative breast cancer
ER: Estrogen receptor
PR: Progesterone receptor
HER2: Human epidermal growth factor receptor 2
IHC: Immunohistochemistry
FISH: Fluorescence in situ hybridization
HER2_low_: HER2-low
HER2_neg_: HER2-negative
ADC: Antibody drug conjugate
scRNA-seq: Single-cell RNA sequencing
NST: Neoadjuvant systematic therapy
scTCR-seq: Single-cell T-cell receptor-sequencing
EC: Endothelial cell
SMC: Smooth muscle cells
CNV: Copy number variation
DEG: Differential expression gene
GO: Gene Ontology
KEGG: Kyoto Encyclopedia of Genes and Genomes, GSEA, gene set enrichment analysis
Treg: Regulatory T cell
DC: Dendritic cell
