# Supplementary material for: Clinical and biological heterogeneities in triple-negative breast cancer reveals a non-negligible role of HER2-low
Source: Breast Cancer Res. 2023 Mar 30;25:34. doi: 10.1186/s13058-023-01639-y (PMC10061837; doi:10.1186/s13058-023-01639-y)
Supplement: Supplementary file 1 — Additional file 1: Fig. S1. Showing the single-cell landscape of all TNBC samples in this study. [file 13058_2023_1639_MOESM1_ESM.pdf]

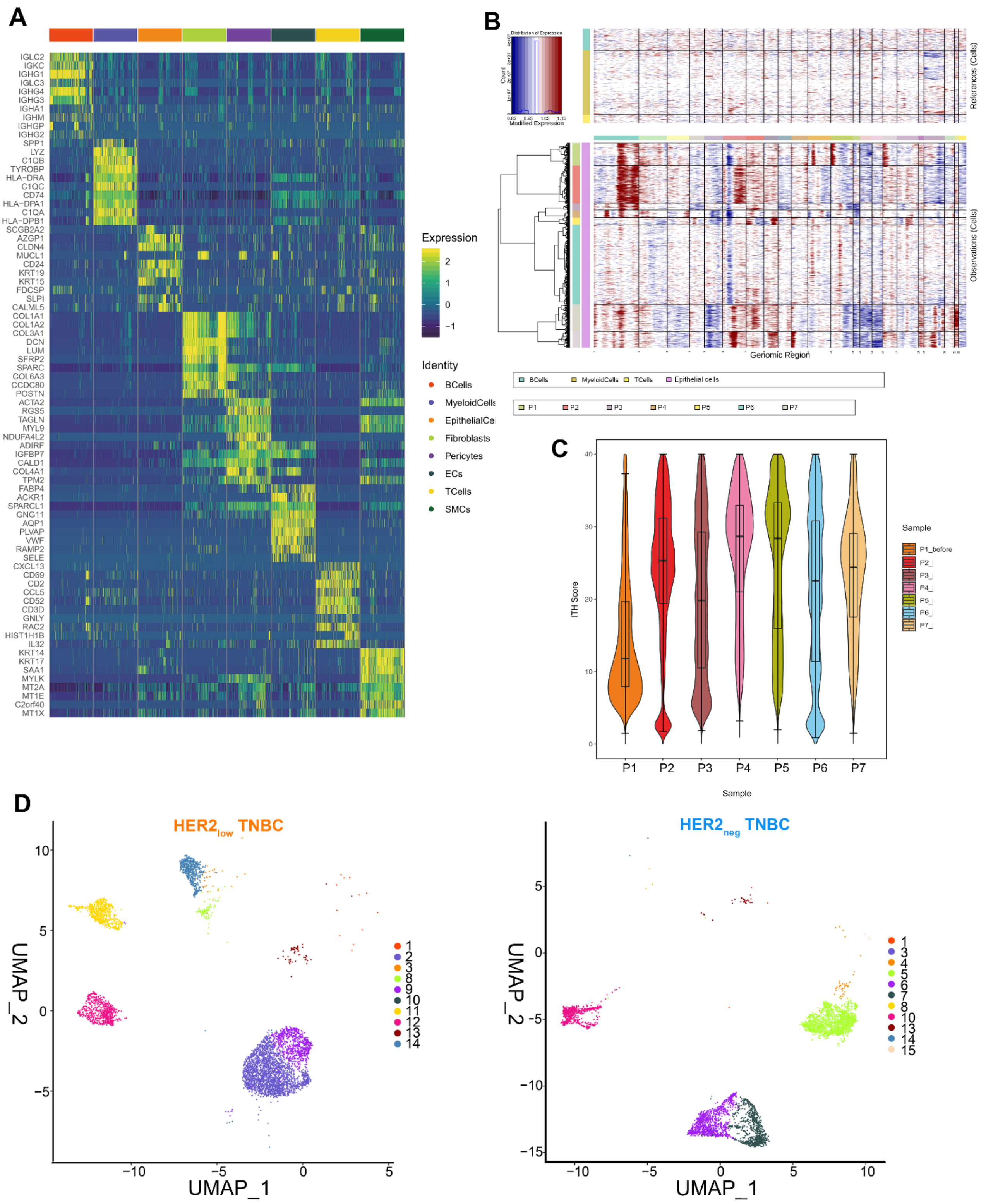

**Figure S1 - Single-cell landscape of all TNBC samples in this study.**

A Heatmap of the top 10 unique gene markers expressed in eight different cell categories. The detailed demographic data are shown in Table S1.

B CNV heatmap of epithelial cells generated by inferCNV. Expression values for normal cells are plotted in the top heatmap, and tumor cells are plotted in the bottom heatmap. The genes are arranged from left to right on the entire chromosome. Chromosomal amplifications are shown as red blocks, and chromosomal deletions are shown as blue blocks.

C ITH scores of the 7 primary TNBC patients.

D UMAPs shows different subclusters of tumor cells contained in the two TNBC groups with different status.
