# Supplementary material for: Clinical and biological heterogeneities in triple-negative breast cancer reveals a non-negligible role of HER2-low
Source: Breast Cancer Res. 2023 Mar 30;25:34. doi: 10.1186/s13058-023-01639-y (PMC10061837; doi:10.1186/s13058-023-01639-y)
Supplement: Supplementary file 2 — Additional file 2: Fig. S2. Showing the UMAPs of tumor cells based on different patients and the expression of four key biological functions in different tumor clusters which suggests HER2neg TNBC and HER2low TNBC harbor distinct tumor biological properties. [file 13058_2023_1639_MOESM2_ESM.pdf]

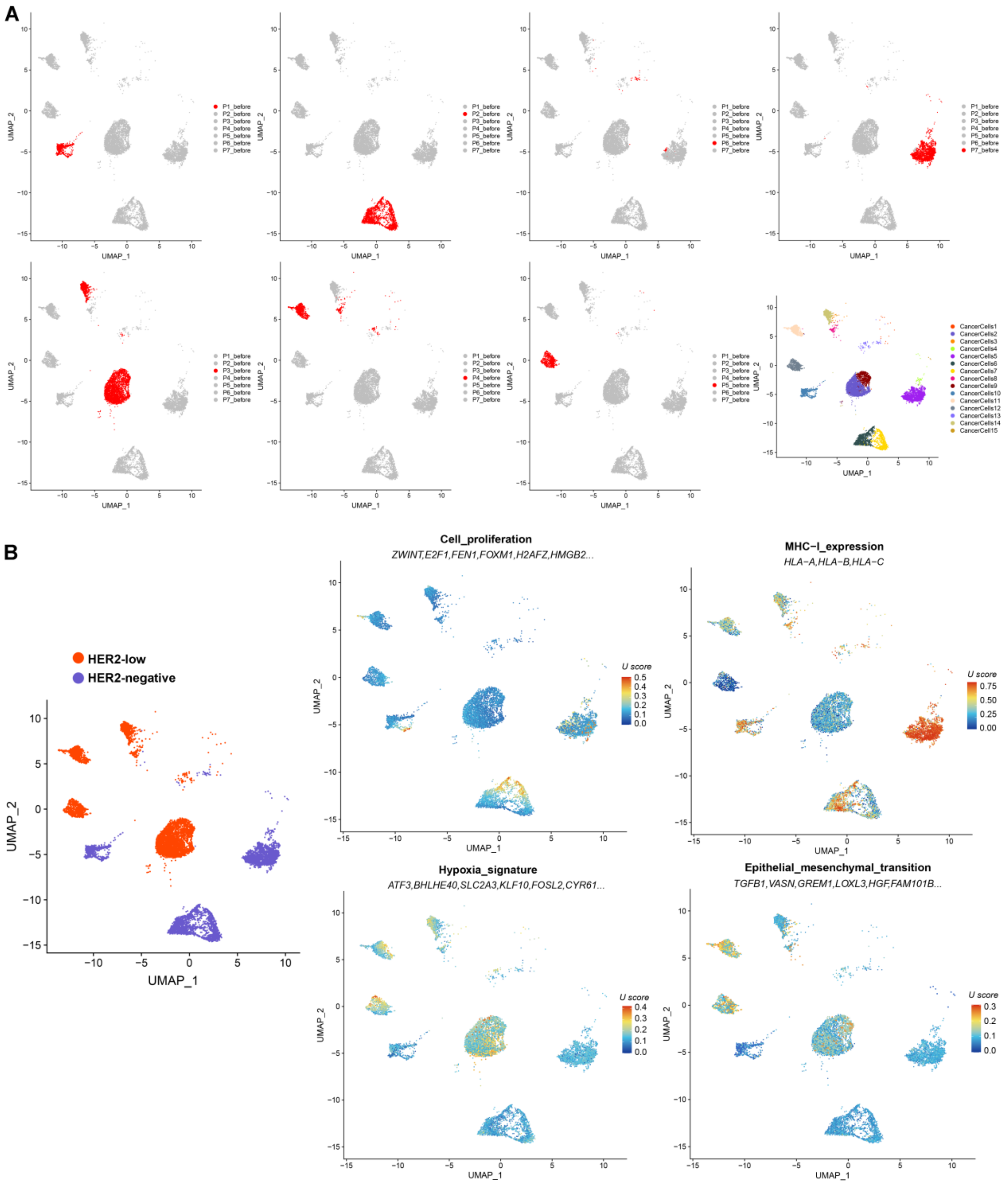

Figure S2 - Tumor cells in HER2$_{neg}$ TNBC and HER2$_{low}$ TNBC harbor distinct biological properties.

A U-MAPs of tumor clusters in different TNBC patients with different HER2 status. HER2$_{neg}$ TNBC: P1, P2, P6 and P7; HER2$_{low}$ TNBC: P3, P4 and P5.

B Assessment of the expression of four key biological functions in different tumor clusters showing that HER2$_{neg}$ TNBC and HER2$_{low}$ TNBC have different tumor properties.
