# Supplementary material for: Clinical and biological heterogeneities in triple-negative breast cancer reveals a non-negligible role of HER2-low
Source: Breast Cancer Res. 2023 Mar 30;25:34. doi: 10.1186/s13058-023-01639-y (PMC10061837; doi:10.1186/s13058-023-01639-y)
Supplement: Supplementary file 3 — Additional file 3: Fig. S3. Showing tumor cell subclusters with different differentiation status had distinct features and ERBB2 levels. [file 13058_2023_1639_MOESM3_ESM.pdf]

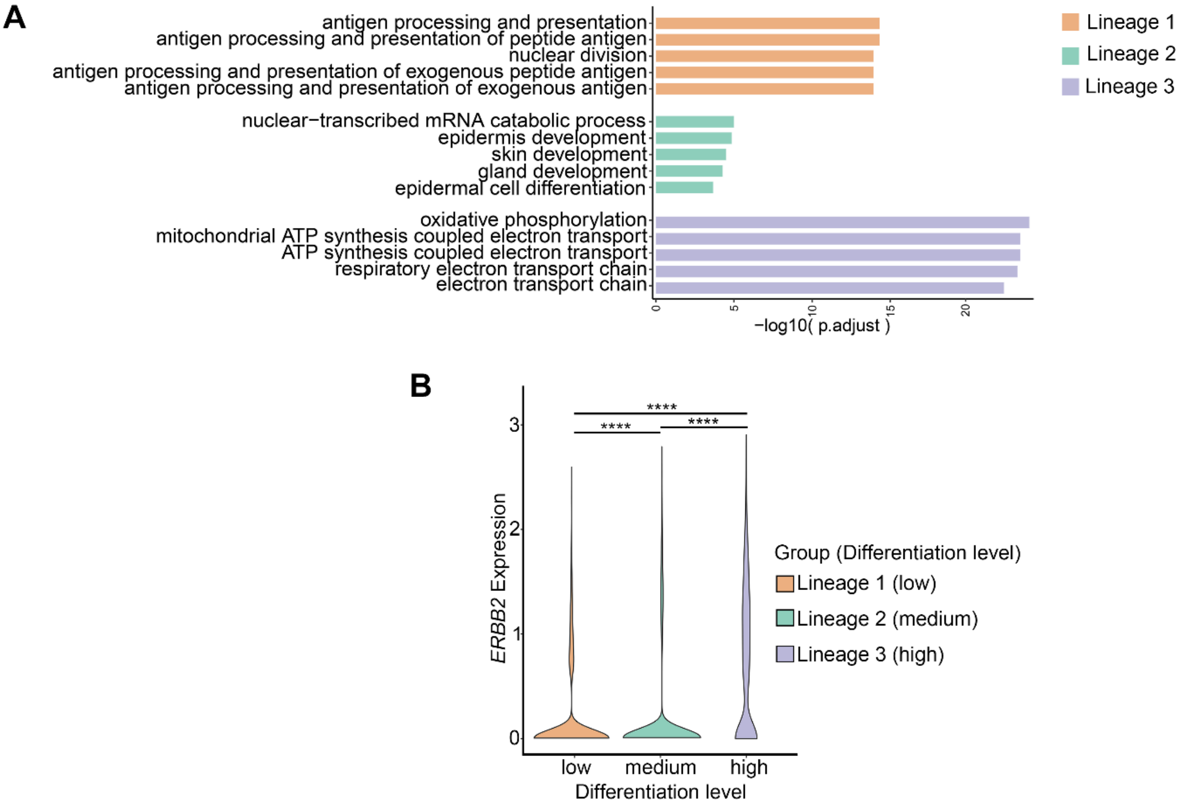

Figure S3 - Tumor cell subclusters with different differentiation status had distinct features and *ERBB2* levels.

A Go enrichment analysis of the three groups of tumor cells with different levels of differentiation in TNBC.

B Violin plot depicts the expression level of *ERBB2* in three groups of tumor cells with different levels (low, medium and high) of differentiation in TNBC, showing that the higher expression level of *ERBB2*, the higher differentiation stage of tumor cells. ****, $P < 0.0001$.
