# Supplementary material for: Clinical and biological heterogeneities in triple-negative breast cancer reveals a non-negligible role of HER2-low
Source: Breast Cancer Res. 2023 Mar 30;25:34. doi: 10.1186/s13058-023-01639-y (PMC10061837; doi:10.1186/s13058-023-01639-y)
Supplement: Supplementary file 4 — Additional file 4: Fig. S4. Showing GO enrichment pathways of highly expressed genes of all tumor cells at three evolutionary states during the pseudotime. [file 13058_2023_1639_MOESM4_ESM.pdf]

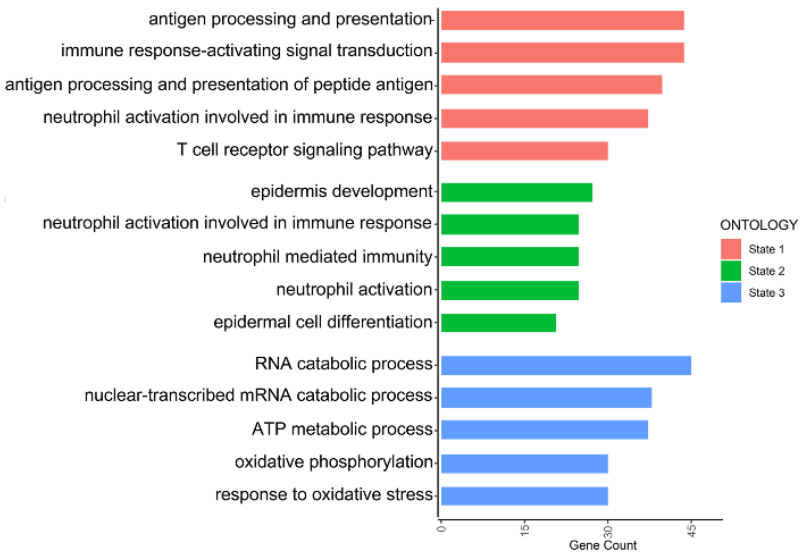

Figure S4 - GO enrichment pathways of highly expressed genes of all tumor cells at three evolutionary states during the pseudotime.

Shown are BP enrichment terms.
