# Supplementary material for: Clinical and biological heterogeneities in triple-negative breast cancer reveals a non-negligible role of HER2-low
Source: Breast Cancer Res. 2023 Mar 30;25:34. doi: 10.1186/s13058-023-01639-y (PMC10061837; doi:10.1186/s13058-023-01639-y)
Supplement: Supplementary file 5 — Additional file 5: Fig. S5. Showing the expression characteristics of AR and AR target genes in the pseudotime trajectory. [file 13058_2023_1639_MOESM5_ESM.pdf]

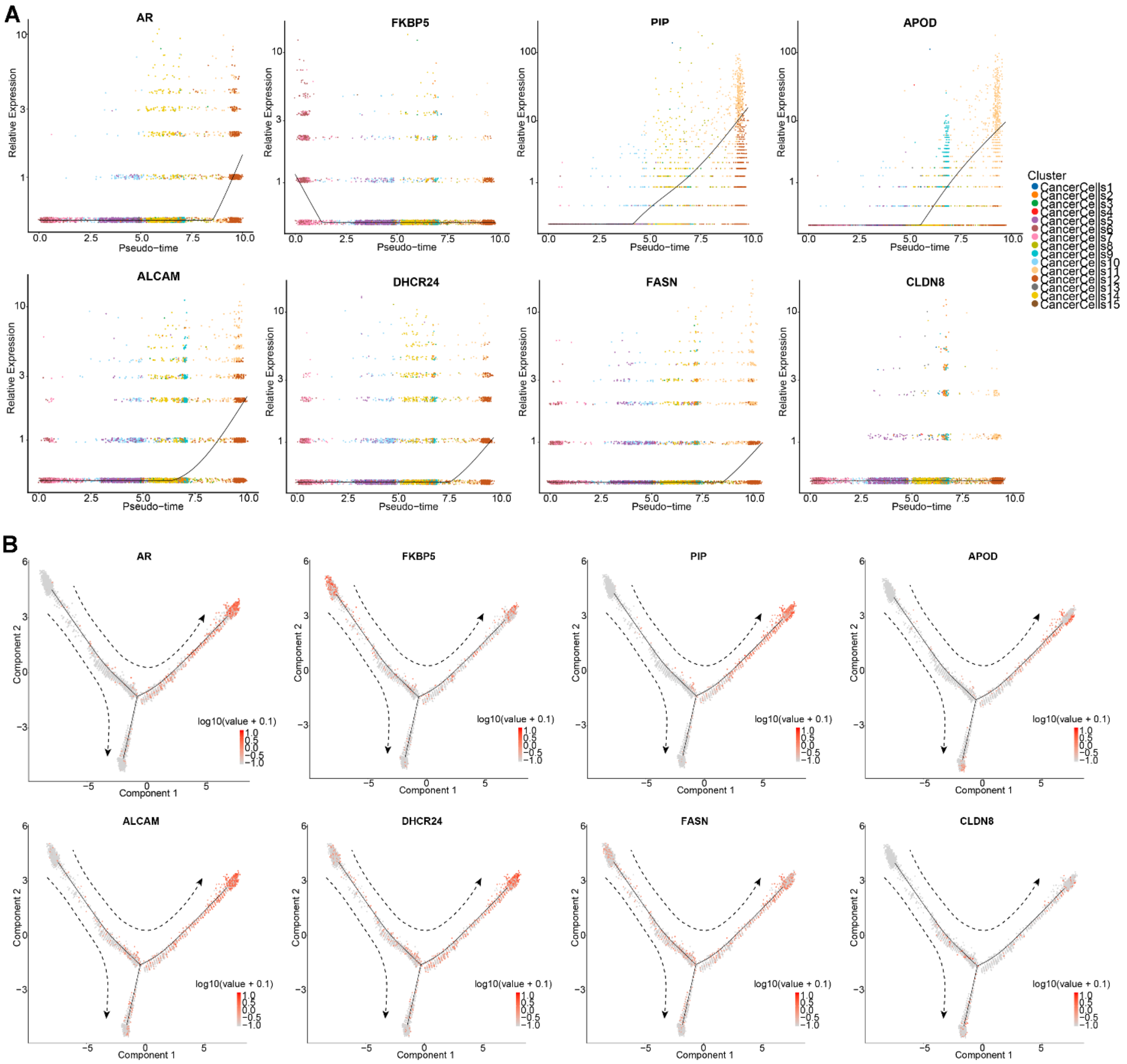

Figure S5 - The expression characteristics of AR and AR target genes in the pseudotime trajectory.

A Changes in the relative expression levels of AR and AR target genes over pseudotime.

B The expression of AR and AR target genes along the pseudotime trajectory. The arrows represent the direction of the pseudotime trajectory.
