# Supplementary material for: Clinical and biological heterogeneities in triple-negative breast cancer reveals a non-negligible role of HER2-low
Source: Breast Cancer Res. 2023 Mar 30;25:34. doi: 10.1186/s13058-023-01639-y (PMC10061837; doi:10.1186/s13058-023-01639-y)
Supplement: Supplementary file 6 — Additional file 6: Fig. S6. Showing different hallmarks of tumor cells in HER2low TNBC and HER2neg TNBC. [file 13058_2023_1639_MOESM6_ESM.pdf]

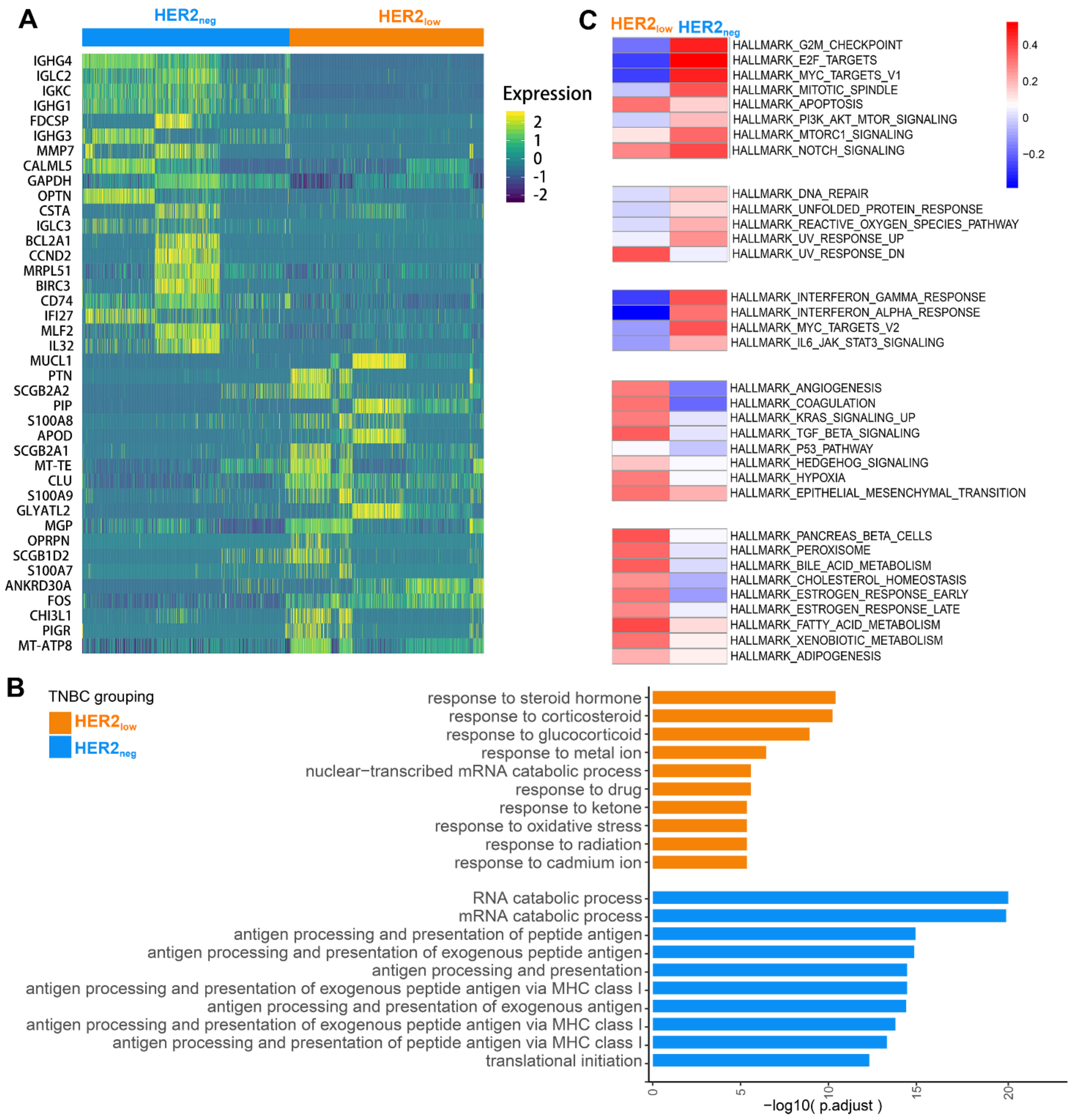

Figure S6 - Different hallmarks of tumor cells in $HER2_{low}$ TNBC and $HER2_{neg}$ TNBC.

A Heatmap of the top 20 highly expressed genes of tumor cells in $HER2_{low}$ TNBC and $HER2_{neg}$ TNBC.

B Heatmap shows the divergence in pathway activities between the two TMBC groups scored by GSVA per cell. Shown are GSVA scores from a lineal model.
