# Supplementary material for: Clinical and biological heterogeneities in triple-negative breast cancer reveals a non-negligible role of HER2-low
Source: Breast Cancer Res. 2023 Mar 30;25:34. doi: 10.1186/s13058-023-01639-y (PMC10061837; doi:10.1186/s13058-023-01639-y)
Supplement: Supplementary file 7 — Additional file 7: Fig. S7. Showing different immune microenvironments of HER2low TNBC and HER2neg TNBC. [file 13058_2023_1639_MOESM7_ESM.pdf]

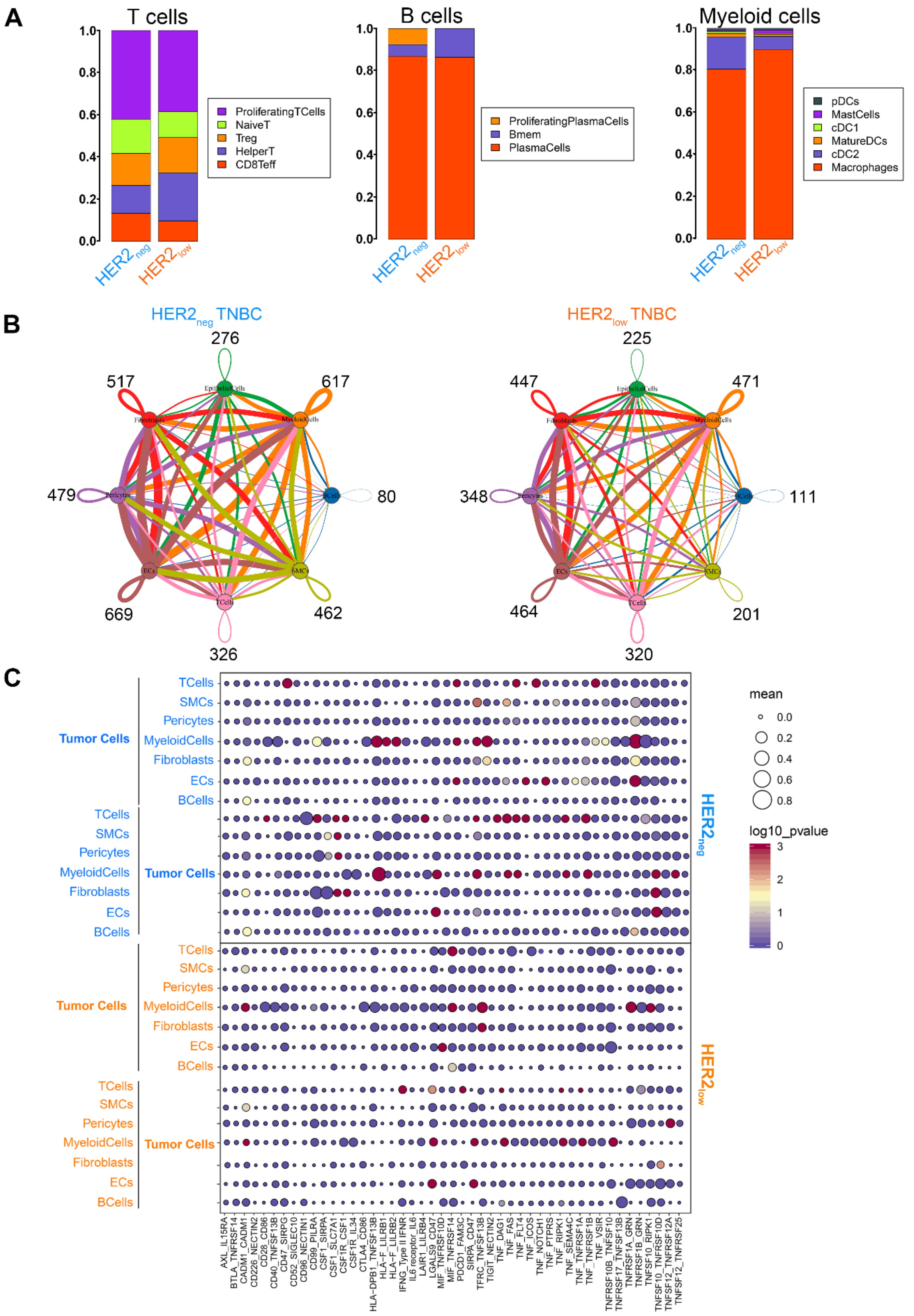

Figure S7 - Different immune microenvironment of $HER2_{low}$ TNBC and $HER2_{neg}$ TNBC.

A Bar plot shows the cellular composition of three types of immune cells in the two TNBC groups.

B Network diagrams show the intercellular communication capacity between cells in two TNBC groups. The network nodes represent various cell types; and the color of each line indicates the ligand (marker) expressed by the cell population of the same color. The line thickness shows the total amount of ligands, and the numbers indicate the number of ligand-receptor pairs connected per cell.

C Overview of selected ligand–receptor interactions associated with immune response between tumor cells and other seven cell types in the two TNBC groups, respectively. Mean interaction levels are indicated by

circle size.
